# Looking for effects of qualia on event-related brain potentials of close others in search for a cause of the similarity of qualia assumed across individuals

**DOI:** 10.12688/f1000research.5977.3

**Published:** 2018-06-04

**Authors:** Sheila Bouten, Hugo Pantecouteau, J. Bruno Debruille

**Affiliations:** 1 Douglas Mental Health University Institute, Montréal, Québec, H4H 1R3, Canada; 2 École Normale Supérieure de Lyon, Lyon, 69007, France; 3 Department of Neurology and Neurosurgery, McGill University, Montréal, Québec, H3A 2B4, Canada; 4 Department of Psychiatry, McGill University, Montréal, Québec, H3A 1A1, Canada

**Keywords:** Really direct brain-to-brain communications, Mind-to-mind Qualia, Consciousness, Event-related brain potentials (ERPs), P3b-P600, IAPS stimuli

## Abstract

Qualia, the individual instances of subjective conscious experience, are private events. However, in everyday life, we assume qualia of others and their perceptual worlds, to be similar to ours. One way this similarity is possible is if qualia of others somehow contribute to the production of qualia by our own brain and vice versa. To test this hypothesis, we focused on the mean voltages of event-related potentials (ERPs) in the time-window of the P600 component, whose amplitude correlates positively with conscious awareness. These ERPs were elicited by images of the international affective picture system in 16 pairs of friends, siblings or couples going side by side through hyperscanning without having to interact. Each of the 32 members of these 16 pairs faced one half of the screen and could not see what the other member was presented with on the other half. One stimulus occurred on each half simultaneously. The sameness of these stimulus pairs was manipulated as well as the participants’ belief in that sameness by telling subjects’ pairs that they were going to be presented with the same stimuli in two blocks and with different ones in the two others. ERPs were more positive at all electrode subsets for stimulus pairs that were *in*consistent with the belief than for those that were consistent. In the N400 time window, at frontal electrode sites, ERPs were again more positive for inconsistent than for consistent stimuli. As participants had no way to see the stimulus their partner was presented with and thus no way to detect inconsistence, these data might reveal an impact of the qualia of a person on the brain activity of another. Such impact could provide a research avenue when trying to explain the similarity of qualia across individuals.

## Introduction

Colors, sounds and smells do not exist in the outside world. They are the creations of our brain in response to light waves, rhythmic variations of air pressure and inhaled molecules, respectively. External stimulations are responsible for action potentials in the brain whose processing may then produce colors, sounds and smells. These, so-called qualia ^1, 2^ are then apparently projected outside around us and constitute our perceptual world, sometimes called the phenomenal world ^3^. Although perceived internally, except in the case of out-of-body experiences ^4^, events, such as feelings of meanings, as in the tip of the tongue phenomenon, or such as emotions, conscious intentions to act and sensations of our body can also be seen as qualia.

Understanding consciousness as consisting of self made qualia leads to one of the most enduring philosophical questions: Are qualia similar across individuals? In other words, is the yellow produced by the brain of one person similar to the yellow produced by the brain of another person? Surprisingly, there is no way to know for sure. The fact that the same word is used by all the people speaking a language to designate a qualia merely establishes a correspondence. It does not prevent the qualia it indicates from varying across these people. The yellow qualia for one person could, for instance, be the blue qualia for another person. Nevertheless, such differences across individuals appear unlikely since many use the same associations and agree that red is a warm color and that blue relates to sadness. In the auditory modality, many associate high pitch sounds with sharpness. Moreover, we use the same metaphor and define these sounds as “high” whereas those of longer wavelengths are said to be “low” sounds (for other metaphors see for instance Lakoff and Johnson ^5^). The same relations between qualia thus appear to exist across people whereas if qualia were different across individuals it seems that these relations should also differ. It could, nevertheless, be argued that metaphors used in a language convey relationships between certain qualia and are thus responsible for building the links between them. However, a number of new metaphors can be understood at their first occurrence ^6^, which suggests that relations between qualia are, at least partly, independent of language.

In any case, if the qualia produced by our brains in response to a given stimulus were not similar across individuals, one could call the entire human race delusional since we all go through our everyday lives and interact with others as if they perceive the world in pretty much the same way as we do. As a matter of fact, if the phenomenal world of each individual were unique, the most fundamental social consensus would be lost. Sharing feelings by verbalizing emotions would be an illusion and our use of language as if each word designates the same qualia would be incorrect. It thus appears reasonable to hypothesize that qualia are similar across individuals and that we are actually living in similar phenomenal worlds.

At first sight, it is tempting to say that qualia could be similar because of the resemblances existing between the brains of humans. However, this idea is questionable for several reasons. First, when macroscopically comparing the brain of people, one can be stricken by the large differences existing between their shapes (with some extreme, such as the one described by Feuillet *et al.* ^7^). There are also problems at the microscopic level. For instance, nothing has been found that distinguishes the so-called color-cells of V1 for blue from the V1 color cells for yellow apart from their afferences ^8^. Thus, applied within a person, the neuronal similarity argument would predict that qualia for blue should be similar to the qualia for red or yellow. Another point can be made with the qualia for white, which is generated by the stimulation of the three types of cone cells, or even by only two complementary colors (e.g., green and red, which stimulate the M and the L cone cells or blue and yellow). How could the V1 « color cells », which are processing the output of these cone cells generate the same qualia? There again, similarities between particular neurons and qualia do not work. So the hypothesis of a similarity of qualia creates a problem. How could qualia be similar across individuals when they are said to be, by nature, totally private events not strictly dependent on brain similarities?

Another, apparently unrelated, question is: how can qualia within a given person be so qualitatively different from one another while theoretically originating from the same type of neuronal bioelectrical activity? Sounds appear to be totally orthogonal to colors or smells. Nevertheless, they are induced by the same depolarizations, such as those induced by Penfield and Jasper ^9^ at different places of the cortex. One way to address this issue is to hypothesize that, while dependent on the well-known bioelectrical activities of neurons, the physical nature of qualia is not limited to these activities. The authors of this second hypothesis can grossly be divided into those suggesting, (a) that qualia are also electromagnetic fields (for a recent review, see Jones ^10^) and (b) those developing the even more controverted theory that qualia also include modulations of the wave function described by quantum mechanics (e.g., 11). Each of these two theories thus introduces phenomena, which, by the immense variety of the instances they include, could provide ways to account for the qualitative differences existing between percepts.

Interestingly, thinking about qualia in terms of electromagnetic fields or in terms of modulations of the wave function could also provide a hint as to how qualia are apparently projected to form our perceived environment and also how they could be similar across individuals while being “private events”. Indeed, both physical phenomena propagate. They can thus be projected and travel between individuals. Therefore, some kind of inter-subjective sharing could theoretically occur. In other words, experiencing a qualia might have an impact on the qualia of another person. This means that, at least in some conditions, the brain activity of a person might be influenced by the activity of the brain of another person. However, to the best of our knowledge, no study has yet *reliably* reported direct and natural brain-to-brain communications. The only report ^12^ we know of did not pertain to spontaneous phenomena either.

Testing this possibility was the first aim of the present study. To achieve this goal, we focused on one operational hypothesis: the event-related brain potentials elicited by a stimulus in one person, particularly the P600, could depend on the stimulus displayed to another person. This P600 is a late event-related brain potential (ERP) elicited by the presentation of meaningful stimuli, such as, words, objects, faces and scenes. It belongs to the P3b family of components despite its late maximum, which occurs around 600 ms post stimulus onset when using complex stimuli such as words, objects, faces and even a little later when using scenes. The greater the amount of new information placed in working memory, the larger the amplitude of this potential ^13, 14^. When a stimulus is unexpected, or when any of its aspects (e.g., its exact time of occurrence) are unpredicted, it elicits a larger P3b-P600 than when the stimulus and every of its aspects are fully predicted. A very large number of cognitive factors can have an impact on the amplitude of the P3b-P600 (see in 14 for the P3b and in 15 for the P600). Thus, if new information was coming from the brain of another person and was having an impact on the activity of the brain of a subject, it might modulate the amplitude of this potential. For instance, if subjects believed that the other person was going to be presented with the same stimulus as them whereas the current information coming from the brain of that person is not consistent with this prior belief, there could be a need for some updating. Accordingly, the P3b-P600s might differ from the ones obtained in conditions in which the information coming from the brain of this other person is consistent with the prior belief.

Exploring pairs of participants and manipulating their beliefs allows the testing of this operational hypothesis. In the present study, a statement announced the two participants of each of these pairs whether or not they would be simultaneously presented with the same stimulus at the beginning of each of the 4 stimulus blocks that were used. These two statements were true in two blocks and false in the two others. If some specific information could pass from the brain of a participant to the brain of the other member of the pair and if this information is not consistent with the belief created by the block statement, it might induce an updating and modulate P3b-P600s. If this were the case, then the existence of direct and spontaneous brain-to-brain communications would be demonstrated if, and only if, that there is no way for participants to see the stimulus the other person is presented with and thus no way to know whether it is consistent with the statement or not.

The second aim of the study had no relation whatsoever to the exploration of the causes of the assumed similarity of qualia across individuals. It was totally separate from the possibility of an impact of one’s activity on the brain of another person. This second goal was to evaluate the impact of social cognition on memory. Indeed, having a mental representation of a partner going through an event (i.e., the presentation of a stimulus), in addition to having a representation of oneself going through the same event, might enrich the encoding in episodic memory and facilitate delayed recognition. Thus, subjects were told to remember each image because there would be a memory test at the end. Our second operational hypothesis was that they would have a higher rate of recognition for the stimuli they were presented with when they *believed* they were seeing the same stimuli as their friend and a lower rate of recognition for the other stimuli. There were thus two independent experimental variables in the present study, consistency for the first (i.e., the brain-to-brain) hypothesis and belief for this second social cognition hypothesis.

## Method

### Participants

Thirty-two right-handed participants (25 F, 7 M), pairs of friends, couples, or siblings were recruited because it was assumed, for this first attempt, that testing people in a close relationship could only increase the odds of natural brain-to-brain communications. The 32 subjects of the 16 pairs underwent exactly the same procedure. All participants learned about the experiment through classified ad websites. They spoke fluent English, were between eighteen and thirty years of age (mean = 23.1, SD = 3.4) and had completed, or were in the process of completing, a university degree. They had normal or glasses-corrected to normal vision. Participants were excluded if they consumed more than twelve drinks of alcoholic beverages per week or if they used recreational drugs, except if they used marijuana less than once per week. Participants were also excluded if they had a history of psychiatric disorder, took medication related to such a disorder, or if one of their first degree relatives had a history of schizophrenia or bipolar disorder. All these inclusion- and exclusion-criteria were checked by an eligibility questionnaire.

### Consent

The two participants of each pair came to the lab together for approximately three hours. Each participant read and signed an informed consent form accepted by the Douglas Institute Research and Ethics Board, which focused on the second aim. This board, which follows the principles expressed in the declaration of Helsinki, also approved the experiment (Douglas REB #12/12). Data were anonymized, which did not distort scientific meaning.

### Stimuli

Stimuli were images selected from the International Affective Picture System (IAPS, 16). Using our own judgment, we chose 560 pictures of this set, including many striking ones, to ensure the maintenance of participants’ attention during the tasks. The experiment consisted first of the study phase, which included four blocks, and then of a memory test phase. As presented in Table 1, which explains their acronyms, each of the four blocks of the study phase, DBd, SBs, SBd and DBs, corresponded to a particular sameness and belief condition. The order of presentation of these four blocks was randomized across subject pairs using a Latin square. We used four different sets of 70 IAPS stimuli. The allocation of each set to each block was also randomized across subject pairs. In study phase blocks in which different pictures were seen by each member of a pair (i.e., in the DBd and DBs blocks), the picture set seen by one participant in DBd was seen by the other participant in DBs, and vice versa. Therefore, all pictures of the four sets were seen by both participants during the study phase. The memory test phase consisted of a fifth set of pictures that contained, in a random order, all the pictures of the study phase mixed with 280 additional pictures.

**Table 1.** Study phase conditions.

Name of study phase condition (acronym). Consistency.	Statements (between quotes) seen simultaneously by the two members of each pair on their own half of the screen before each block-condition of the study phase and what reality was.
Different Believe-different (DBd). Consistent.	“Try to remember the next 70 pictures. You will now see different pictures than your friend,” and they did see different images.
Same Believe-same (SBs). Consistent.	“Try to remember the next 70 pictures. You will see the same pictures as your friend,” and they did see the same pictures.
Same Believe-different (SBd). Inconsistent.	“Try to remember the next 70 pictures. You will see different pictures than your friend,” but they saw the same pictures.
Different Believe-same (DBs). Inconsistent.	“Try to remember the next 70 pictures. You will see the same pictures as your friend,” but they saw different pictures.

### Procedure

**Figure 1.**
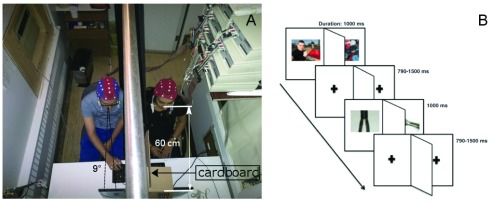
**A**. Experimental setup. Note that the black “cardboard” arrow is above a beige horizontal rectangle. This rectangle is a piece of cardboard whose rear edge is stuck on the upper edge of the computer screen. It includes a notch within which is inserted the vertical piece of cardboard that separates this computer screen into two halves. This prevents participants from seeing the stimulus presented to his/her partner. The tip of the black arrow coincides with that vertical piece. The “9°” indicate the visual angle encompassed by the stimulus presented to each partner, whose eyes are about 60 cm from the computer screen. **B**. IAPS stimulus presentations for each of the 4 conditions of the study phase. The different-and-believed-different (DBs) condition is used as an example. Note again the division of the screen into two halves by a vertical cardboard piece, preventing subjects from seeing each other’s stimuli, but not from feeling close to one another.

The study phase ( Table 1) was followed by the memory test phase. As illustrated by Figure 1B, each stimulus of the study phase was presented for 1000 ms and was followed by a white screen with a black fixation cross, the duration of which randomly varied between 790 and 1500 ms to prevent the development of a contingent negative variation ^17^. Participants could see their partner in their very peripheral vision field without moving their eyes. Nevertheless, even if they moved their eye or move their heads they could not see the part of the screen their partner was watching ( Figure 1A illustrates this unusual setting). Participants were told to look at each picture for the subsequent memory test phase. Stimuli in that latter phase were presented for 3000 ms in order to allow time for participants to respond.

During the memory test phase, participants were required to respond by pressing keys on a *shared* computer keyboard. The participant seated on the left hand side of the keyboard used the typewriter keys and pressed ‘1’ to indicate (s)he believed to have seen the picture previously, and ‘2’ to indicate (s)he believed not to have seen the picture previously. The participant seated on the right hand side of the keyboard used the numeric keypad and pressed ‘4’ to indicate (s)he believed to have seen the picture previously, and ‘5’ to indicate (s)he believed not to have seen the picture previously.

At the end of the memory test phase, there was a debriefing session where participants were asked 4 questions, mainly designed to explore attention differences and whether they detected any deception. The first was: “Did you feel more attentive/distracted seeing the pictures when your friend was present?”. The second was: “Did you feel any different when you knew your friend/partner/relative was looking at the same images that you were seeing? ». The third was: « Did you feel any different when you knew your friend/partner/relative was looking at different images than you were seeing? ». The fourth was: « Did you feel deceived at any point during the experiment? ».

### Data acquisition

Behavioral key presses were recorded during the memory test phase, as well as the verbatim of the response to the debriefing session’s questions. The electro-encephalogram was recorded from 28 electrodes mounted in an elastic cap (Electro-Cap International) during the study phase. Electrodes were placed according to the modified expanded 10–20 system ^18^. For each participant of each pair, these electrodes were grouped into three subsets: sagittal (Fz, Fcz, Cz and Pz), parasagittal (F3/4, Fc3/4, C3/4, Cp3/4, P3/4, and O1/2), and lateral (F7/8, Ft7/8, T3/4, Tp7/8 and T5/6). There was a separate set of amplifiers for each participant. The right earlobe was used in each subject as the reference for his/her set of amplifiers while the ground was taken from an electrode two centimeters ahead of Fz. For both sets of amplifiers, high- and low-pass filter half-amplitude cut-offs were set at 0.01 and 100 Hz, respectively, using an additional 60 Hz electronic notch filter. EEG signals were amplified 10,000 times and digitized online at a 256 Hz sampling rate and stored in a single file with 56 (28 × 2) channels.

### Data processing and measures

In each trial, electrodes contaminated by eye movements, excessive myogram, amplifier saturations or analog to digital clipping were removed offline by setting automatic rejection criteria. Electrodes for which analog to digital clipping exceeded a 100 ms duration and electrodes for which amplitude exceeded +/- 100 mV were discarded when these excesses were within the -200 to +1000 ms. The baseline was set prior to the onset of the stimulus, from -200 to 0 ms. Averages were calculated for each block and each subject in a 1400 ms time window, beginning 200 ms before the onset of the stimulus and lasting for 1200 ms after the stimulus onset. Following averaging, each file was divided into two files, each containing the ERPs of a single subject. The ERPs of each of the 32 subjects for each consistency-with-belief condition (consistent vs. inconsistent) and each belief condition (belief-same vs. belief-different) were then computed and measured independently of the pair of participants they initially belong to. Based on our a priori hypothesis, we focused on the late positive component (LPC or P600) and computed the mean voltages of ERPs in the 650–950 ms time window for all electrodes, all subjects and all four conditions. Nevertheless, because visual inspection detected a small difference in the time window of the potential that preceded the P600, that is, the N400, mean voltages were also measured in the 350–550 ms time window to explore these differences and create a priori hypotheses for future studies.

### Analyses

Repeated-measures ANOVAs were run with the version 20 of the IBM-SPSS software package to analyze these measures using a multivariate approach. For the sagittal subset of electrodes, they had consistency of the actual stimuli with the belief (consistent vs. inconsistent) or social cognition (belief that stimuli were the same vs. belief they differed) and electrodes as within-subject factors. For parasagittal and lateral electrodes, a fourth within-subject factor, hemiscalp (right vs left), was included. Given that there was only one group of 32 subjects, there was not any between-subject factor. The Greenhouse and Geisser ^19^ procedure was used when required to compensate for heterogeneous variances, in which case the original F values and the corrected p values will be given. To provide a priori hypotheses for future studies, we also completed one-way ANOVAs at each electrode to assess each effect found.

## Results

### Electrophysiological results: Testing the first hypothesis of natural brain-to-brain communications

**Figure 2.**
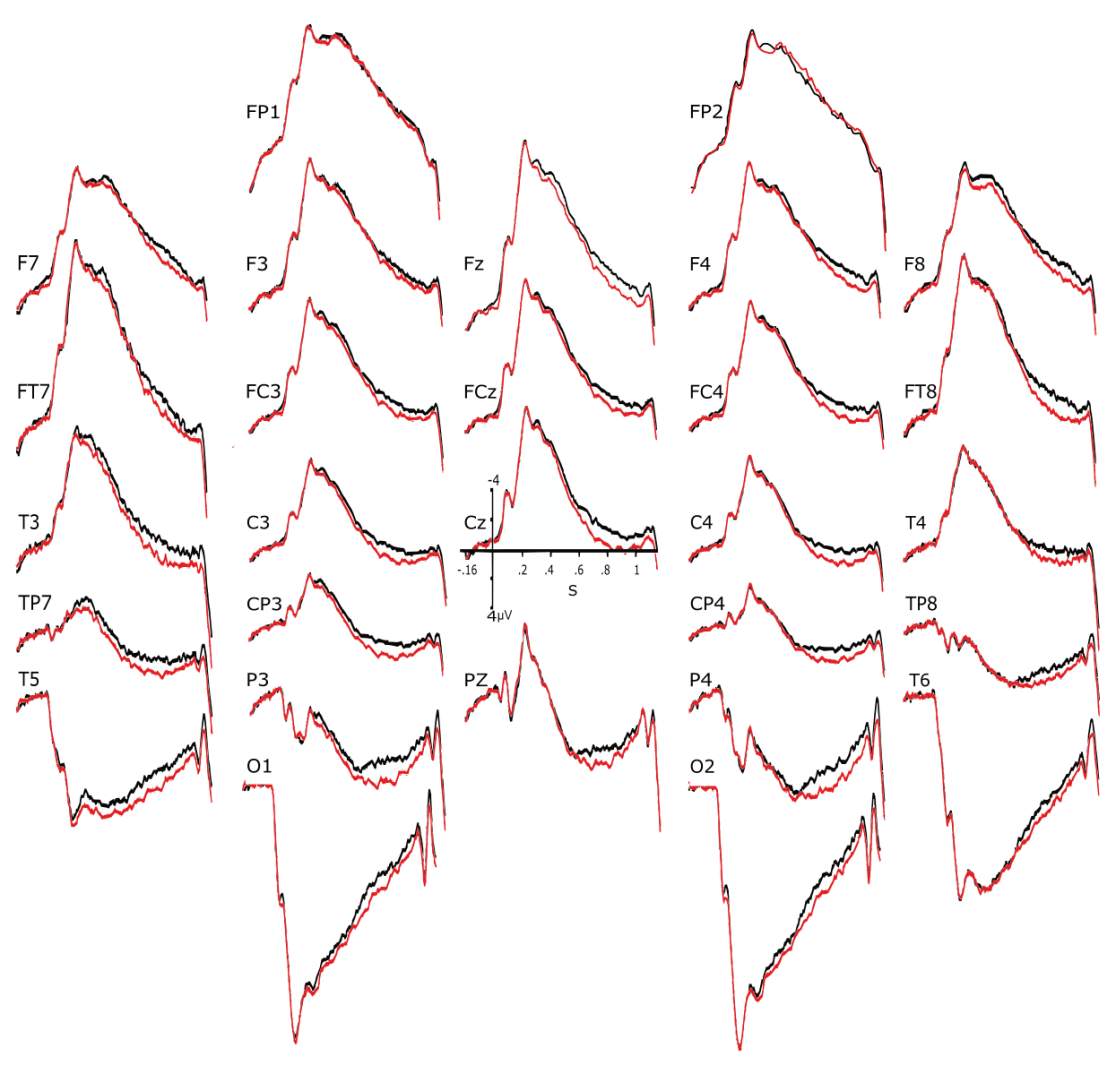
ERP effects of consistency Grand average (n = 32) of the event-related brain potentials elicited by the stimuli of the international affective picture system (IAPS). Black waveforms are for the stimulations (140 in each consistency condition) that were consistent with the belief of the participants. This includes the block-condition where the stimulus presented to one partner differed from the stimulus simultaneously presented to the other partner while these partners believed the two stimuli differed. It also includes the block-condition where the two stimuli were the same and believed to be the same. Red waveforms are for opposite stimulations (also 140 for each partner) where the actual sameness of the two stimuli was inconsistent with the participants’ belief. The downward notch at the end of most tracings correspond to the off-effect (i.e., to the disappearance of the IAPS picture used as a stimulus), which might also be associated to a vertical eye movement artefact kept since 1000–1200 ms was a non-reject interval.

**Figure 3.**
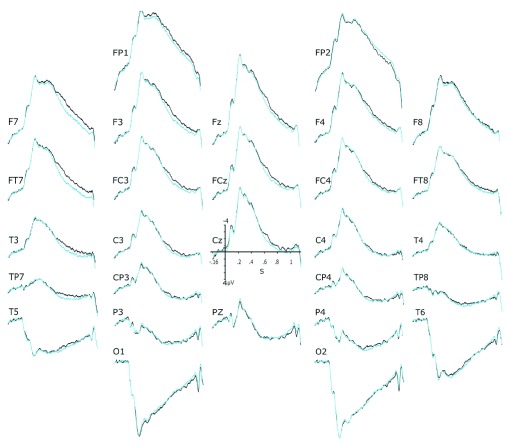
ERP effect of social cognition Grand average (n = 32) event-related brain potentials elicited by the stimuli of the international affective picture system (IAPS). Black waveforms are for an average of the two block-conditions where the participant believed (s)he was seeing stimuli (140 for each partner) different from the ones simultaneously presented to his/her partner. Blue waveforms are for an average of the two block-conditions where the participant believed (s)he was seeing the same stimuli (140 for each partner) as the ones simultaneously presented to his/her partner. The downward notch at the end of most tracings correspond to the off-effect (i.e., the disappearance of the IAPS picture used as a stimulus), which might also be associated to a vertical eye movement artefact kept since 1000–1200 ms was a non-reject interval.

Figure 2 shows the grand averages for the 32 subjects of the 16 pairs tested. Visual inspection of the P600 time window at the electrodes where the amplitude of this ERP component is usually maximal, that is, at the central (Cz) and parietal (Pz) midline sites, suggests slightly larger P600s when pairs of stimuli were inconsistent with the prior belief (red lines) than when they were consistent (black lines). At frontal electrode sites, ERPs were also slightly more positive for inconsistent- than for consistent-stimuli in the time window of the potential that precedes the P600, that is, the N400. Social cognition in itself ( Figure 3) was found to correspond to some small ERPs differences, mainly located at left anterior lateral electrode sites (i.e., F7 & Ft7). There, the prior belief that stimuli seen by the other member of the pair were different from the ones seen by the participant goes with ERPs (black lines) that seem a bit less positive than the ERPs elicited by stimuli of the two blocks preceded by the statement mentioning that the same stimuli will be presented to the two participants (blue lines).

Table 2 includes the significant (p < .05) effects found in the ANOVAs performed on each subset of electrodes in the time-window of the P600. It reveals that the effect of consistency was significant at each electrode subset.

**Table 2.** Statistically significant effects found in the three ANOVAs run with consistency, electrode and hemiscalp as factors in the P600 time windows (650–950 ms).

Electrode Subset	Factors	*F*	*p*
**Sagittal**	Consistency	8.73	.006
	Consistency × Electrode	3.36	.038
Posthoc at Cz	Consistency	10.61	.003
**Parasagittal**	Consistency	8.21	.007
**Lateral**	Consistency	7.49	.010

**Figure 4.**
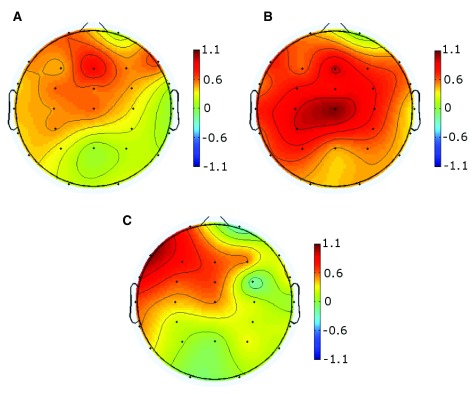
Maps of the ERP effects of consistency and belief. Spline interpolated isovoltage scalp maps computed after subtracting the mean voltages of the grand average ERPs **A**) in the N400 time window of the consistent conditions from the mean voltages of the inconsistent conditions, **B**) same as **A**) but for the P600 time windows, **C**) in the P600 time windows of the belief-different conditions from the belief-same conditions.

In addition, as mentioned, visual inspection of the ERPs according to consistency ( Figure 2) revealed small differences within a time windows preceding that of the P600 (e.g., at Fz). Exploratory ANOVAs were thus run to test the significance of these differences within the 350–550 ms.time window. Consistency was found to interact with electrodes at the sagittal subset of electrodes (F (3-93) = 3.93, p = .029), as illustrated by Figure 4. The post hoc analysis performed at Fz to locate the source of this interaction confirmed the significance of this effect at this frontal sagittal site (F (1-31) = 7.94, p = .008), which is reported here to create a priori hypotheses for future studies, as there was no relation with a priori hypotheses. No main effects of, or interaction with, consistency was found for the two other electrode sets.

**Figure 5.**
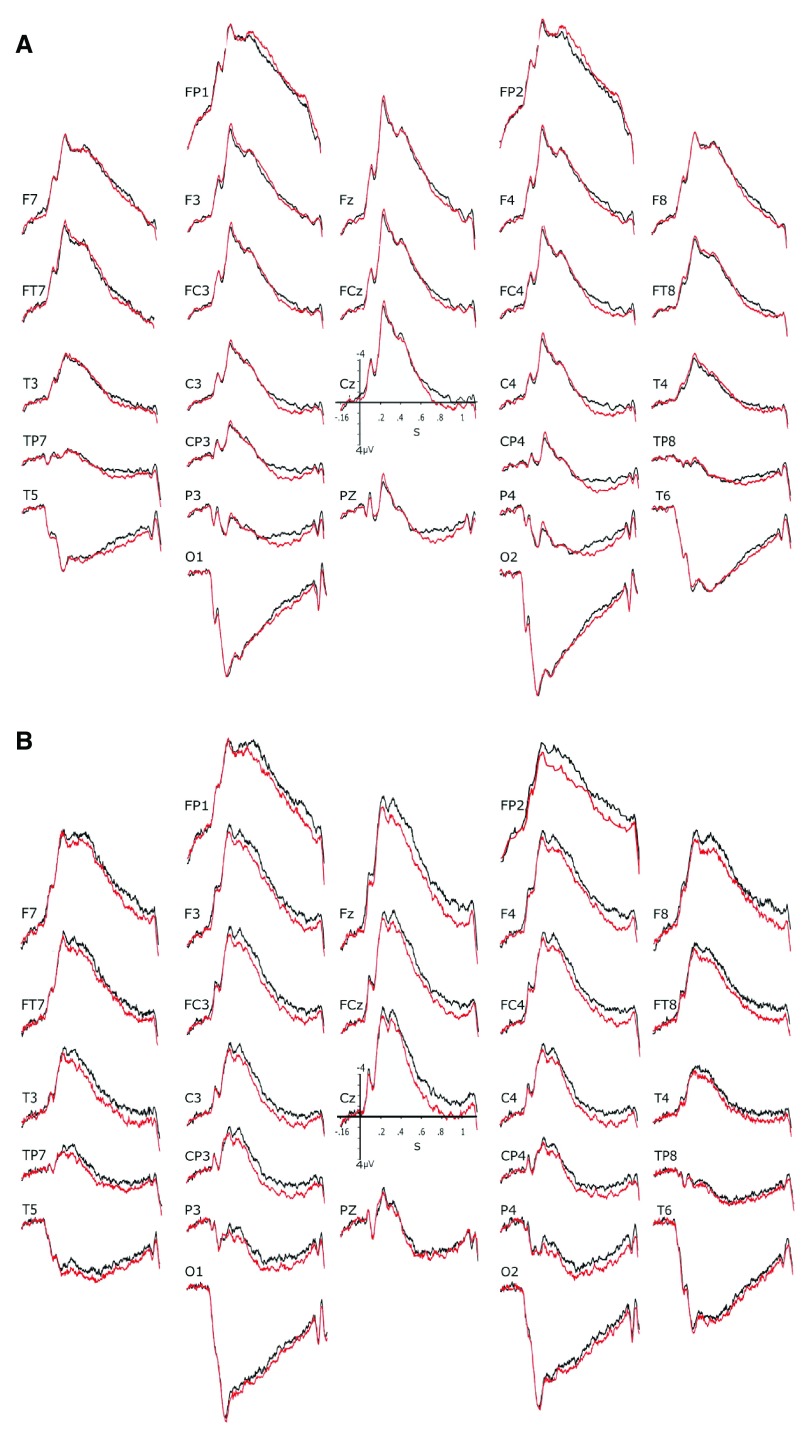
**A**) & **B**) Assessing replicability of consistency effects and eye movements towards the partner. Grand average of the event-related brain potentials (ERPs) of the two consistency conditions for the 16 participants who where on the right side of their partner. Colors as in Figure 2. **B**) same as **A**) but for the 16 participants who were on the left side of their partner. Colors as in Figure 2.

The replicability of these findings was explored by computing grand averages of the 16 subjects who had their partner on their right side and those who had their partner on their left side. Note that these two sets of subjects went through the exact same procedure and conditions except that each of them was hooked up to a particular set of amplifiers. Figure 5A and B display these grand averages. They bring support for replicability to the extent that 1) no difference was found in a direction that would be opposite to that observed in the grand average of the 32 participants and 2) ERPs appeared to be more positive for inconsistent than for consistent conditions in both groups during the P600 time window at central, centro-parietal and parietal electrode sites. Nevertheless, it can be noted that, at frontal sites, these differences appeared to be present only in subjects who had their partner on their right side and that, within the N400 time window, these differences appeared to be restricted to this last subgroup.

Interestingly, these ERPs allow eliminating the possibility of horizontal eye movements that would have been aimed at looking at the partner. Indeed, this were the case, one would have seen differences maximal at F7/F8 between the two subgroups. This is not what the visual inspection of these two figures reveals.

### Electrophysiological results: Testing the social cognition hypothesis

The ANOVA performed on each of the three electrode subsets with social cognition as factor revealed only a significant belief × hemiscalp interaction at the lateral subset (F(1, 31) = 4.68, p = .038). The post hoc run for the left hemiscalp at this subset then showed a significant belief × electrode interaction (F(4, 124) = 3.25, p = .014). To find the source of this interaction, a one-way ANOVA was run at F7, where differences looked the “largest” on Figure 3. It confirmed the existence of a marginally significant effect (F(1, 31) = 5.18, p = .030).

### Behavioral Results: Testing the social cognition effect on the encoding in episodic memory

As shown in Table 3, in the memory test phase, there was no difference between study phase conditions in the number of stimuli correctly recognized (hits) or in the number of misses. In sum, participants did not better recall images from any particular condition of the study phase. Similarly, there was no effect of the condition of the study phase on the reaction times of the memory test phase ( Table 4).

**Table 3.** Average number of hits versus misses by study phase condition.

Study Phase Condition	Number of Hits (SD)	Number of Misses (SD)
SBd	43.3 (10.1)	26.1 (9.7)
DBs	42.5 (10.9)	26.6 (11.1)
SBs	43.4 (12.3)	26.6 (12.2)
DBd	43.3 (10.9)	26.2 (10.8)

**Table 4.** Average reaction time of hits versus misses in milliseconds by study phase condition.

Study Phase Condition	Average Reaction Time of Hits in milliseconds (SD)	Average Reaction Time of Misses in milliseconds (SD)
SBd	1081 (125)	1185 (151)
DBs	1080 (135)	1138 (171)
SBs	1081 (125)	1157 (153)
DBd	1092 (133)	1146 (176)

The results of the debriefing session were as follows. For the question: “Did you feel more attentive/distracted seeing the pictures when you friend was present?”, 8 participants said they were more distracted, 18 said there was no difference, 6 said they were more attentive. To the question: “Did you feel any different when you knew your friend/partner/relative was looking at the same images that you were seeing?”, 17 participants said they felt the same, 14 said they felt different. To the question: “Did you feel any different when you knew your friend/partner/relative was looking at different images than you were seeing?”, 9 said yes, 22 said no. For the fourth question “Did you feel deceived at any point during the experiment?”, 27 said no, 3 misunderstood “deceived”, 2 said yes, but when asked why, they did not suspect the statements about the sameness of stimuli. Their suspicion pertained to other aspects (e.g., one said, after the stimulus presentation computer unexpectedly stopped, “I thought that when the computer crashed it was deliberately done so that it was more difficult to remember”).

Mean voltages of the event-related brain potentials elicited by the IAPS stimuli in the P600 time window This Excel file includes, in each cell, the mean voltage of the ERPs of one participant (lines: sub2 to sub38), for one electrode site (columns C3 to TP8) for consistent and inconsistent conditions. Click here for additional data file. Copyright: © 2018 Bouten S et al. 2018 Data associated with the article are available under the terms of the Creative Commons Zero "No rights reserved" data waiver (CC0 1.0 Public domain dedication).

Mean voltages of the event-related brain potentials elicited by the IAPS stimuli in the N400 time window Same as dataset 1, but for the N400 time window. Click here for additional data file. Copyright: © 2018 Bouten S et al. 2018 Data associated with the article are available under the terms of the Creative Commons Zero "No rights reserved" data waiver (CC0 1.0 Public domain dedication).

Mean voltages of the event-related brain potentials elicited by the IAPS stimuli in the P600 time window for BS and BD conditions Same as dataset 1, but for the conditions in which the participants believed (s)he was seeing the same stimulus as the one simultaneously presented to his/her partner (BS, for believe same) and for the conditions in which they believed it differed (BD, for believe different). Click here for additional data file. Copyright: © 2018 Bouten S et al. 2018 Data associated with the article are available under the terms of the Creative Commons Zero "No rights reserved" data waiver (CC0 1.0 Public domain dedication).

## Discussion

In each recording session of this study, pairs of related participants were tested together. In each trial, two pictures taken from the international affective picture system (IAPS) were presented simultaneously, one for the first participant, the other for the second participant of the pair. All 32 participants of the 16 pairs tested were asked to remember these pictures during the four different blocks of the study phase. These pictures were then presented again, mixed with new ones, during a subsequent memory test phase.

During both phases, the computer screen was divided in two halves that were separated by a vertical cardboard perpendicular to the screen. Each participant of a pair sat in front of one half of the screen and was presented with one picture at a time. There was no way for a participant to see the picture simultaneously presented to the other participant.

Sameness was manipulated. In two of the four conditions of the study phase, participants were presented with the same picture simultaneously (S conditions). They were presented with two different pictures in the two others conditions (D conditions). Social cognition was studied by manipulating the belief (B) pertaining to what the other member of the pair was presented with. Just before the beginning of each condition, or block, of the study phase, participants saw one of two statements on the screen, announcing whether or not the same picture would appear for both of them on each half of the screen. The four conditions of the study phase were thus: different believed-different (DBd), same believed-same (SBs), different believed-same (DBs) and same believed different (SBd), the latter two thus including statements that were inconsistent with reality.

Event-related brain potentials elicited by the IAPS pictures were recorded during these four conditions of the study phase to test our first hypothesis of natural and spontaneous brain-to-brain communications. In accordance with this hypothesis, ERPs were found to be more positive within the P600 time window in the inconsistent than in the consistent conditions at each of the three subsets of electrodes (e.g., sagittal, parasagittal and lateral). Note, however, that then, the independence of participants in the ANOVA would not be achieved and degrees of freedom would have to be reduced. Most importantly, these inter-dependences were found whereas each participant of each pair could not see the stimulus the other subject of the pair was presented with and, thus, could not verify whether or not the statements were true.

Usual interpretations of these ERPs difference could be ruled out. First, because, in contrast with the second hypothesis, there was no effect of the conditions of the study phase on the scores of the memory test phase. The ERP differences found between the conditions of study phase could thus not be related to a Dm effect; that is, to larger P600s at fronto-central electrode sites for stimuli that benefit from a deeper encoding in episodic memory ^20, 21^. Second, the ERP differences found were also unlikely to be related to differential allocations of attentional resources. Indeed, all stimuli had the same task relevance since they equally had to be memorized. Moreover, they could not capture attention differentially, since their use for each of the conditions of the study phase was counterbalanced across pairs of participants.

The statements seen by participants as to whether or not they would be presented with the same stimuli as the other participant of the pair could have theoretically modulated the allocation of attentional resources and thus P600 amplitudes. Nevertheless, these statements could not have had an effect depending on whether or not the stimuli were actually consistent with the statement, since it was something participants had no knowledge of. Third, more preconscious processing does not seem to be useful to account for the greater P600s obtained in the inconsistent than in the consistent conditions. Indeed, why would more processing occur for inconsistent- than for consistent-conditions when all stimuli equally had to be memorized?

On the other hand, participants were side by side and could hear and see each other in their very peripheral visual field (i.e., 90 degrees). Thus, they could in principle influence each other (e.g., through breathing variations, subtle body movements, like postural reactions to aversive stimuli, facial mimicry, eye movements etc). It thus has to be discussed whether or not the present results could be in line with Dumas’ *et al.* work ^22^ on hyperscanning and inter-brain synchrony mediated through the mirror neuron system. Given that our participants did not have any task to perform, other than to look at the stimuli, part of their attention could have been allocated to what their partner was doing. Therefore, we have to ask whether the processing of the partner’s movements could have been responsible for our results. At first, this does not seem impossible since, when participants were not seeing the same stimuli, they might not “be moved” in the same way and their systems might have detected that move difference. However, to account for the results obtained here, the effect of such a detection would also have to depend on the statement. When the subject was told (s)he will be presented with same stimuli, then, the move difference detected should be further processed since it is contradictory information. Nevertheless, ERP results are not in accordance with this interpretation. The unexpected effect found in the N400 time window show that inconsistency of the information with the belief was taken into account and had consequence on stimulus processing. This consequence started as early as the time of onset of the N400 whereas much longer time appears to be necessary for all the necessary steps, which include; the processing of the stimulus, the activation of an appropriate emotional response, the execution of this response, its processing from the very peripheral visual field of the other participant and the detection of its inconsistency with the expectancies of this participant. It thus appears very unlikely that the effect of consistency found here could be due to partner’s moves.

Thus, in accordance with the first hypothesis, the larger P600s in statement inconsistent- than in statement consistent-conditions can be used to support the existence of spontaneous brain-to-brain communications (together with smaller N400s). These communications seem to permit our system to detect that others are not going through what we expected they would go through. The fact that, at the debriefing session, participants did not notice any deception shows that this detection remains unconscious. Thus, although P600s can index conscious process ^13, 14, 23, 24^, the larger P600s obtained in inconsistent- than in consistent-conditions would not index the conscious detection of this kind of differences, in accordance with everyday life, where such things are inscrutable. But the fact that brain-to-brain communications can have such an impact on the P600s is consistent with the possibility of an additional content of consciousness that is neither verbalizable nor distinguishable from the qualia each participant would have had if (s)he were alone. In other words, the spontaneous brain-to-brain communications found could theoretically be responsible for mutual qualia enrichment as hypothesized in the introduction. Qualia of others might contribute to our own by a merging process occurring without our knowledge. However, it is very important to point out that *nothing* in the present data *has* to be related to qualia. The P600 effects of consistency only support the existence of spontaneous and direct brain-to-brain influences that just *could* be related to consciousness.

Nevertheless, only qualia have been related to physical phenomena that propagate. Thus, although this relation is speculative, these are the only physical phenomena that can be discussed as those potentially underlying the direct brain-to-brain influences found. As mentioned, theories have been put forward that relate qualia to electro-magnetic fields, as reviewed by Jones ^10^ and to the collapse of the so-called wave function studied in quantum mechanics, as proposed by Hameroff and Penrose ^11^. According to these ideas, qualia might then be responsible for the natural brain-to-brain communications found. The electromagnetic hypothesis can be based on the sensitivity to magnetic fields of at least two molecules: magnetite, whose presence has been demonstrated in the human brain ^25– 27^, and cryptochrome ^28^. Furthermore, it is consistent with the fact that mammal behaviors have been shown to depend on magnetic fields, such as that of the earth ^29, 30^. However, two properties of magnetic fields are at odds with the idea that the magnetic fields generated by one participant could affect the brain activity of the other participant. First, the magnetic fields generated by the activity of the human brain (only 10 to 10 ^3^ femto Tesla) are much smaller than the magnetic noise of an urban environment (about 10 ^8^ femto Tesla). Second, magnetic fields decrease with the square of the distance. The heads of the two subjects of each pair were separated by about 40 cm, a distance much larger than the distance separating the brain from the devices used to capture the magnetic fields it generates in magneto-encephalography (MEG, i.e., less than one cm). Finally, our ERP recording room was not shielded like a MEG recording room. Urban magnetic noise was thus much more important than any field a human brain can generate. These factors make the electromagnetic field explanation appear less likely. In contrast, our experimental conditions and results seem to be more consistent with the *theories* of consciousness that see qualia as, at least partly, underlain by a modulation of the wave function, and that see direct brain-to-brain communications possible through quantum entanglement [e.g., 31]. Indeed, such modulations do not decrease with distance and could involve many atoms ^32^. Nevertheless, it is pretty clear at this point that only speculations can be made as to the physical nature of the phenomena by which the activity of a brain could have an impact on the activity of another brain.

On the other hand, the finding of this impact raises the problem of irrelevant interferences. Indeed, the activity of many brains could then affect the activity of our own. It appears logical to think that filtering exists to prevent such perturbations. One possibility is that, the close relationship existing between the members of each pair in the present study is a prerequisite for the impact to occur, as it may depend on empathy and/or prior common memories. A further study should thus test whether the inconsistency effect reported here on ERPs could be found with pairs of participants who do not know each other before being tested together. On the other hand, filtering should also operate when the information is irrelevant, for instance when it is redundant with expectations and when the partner is not close by and/or when one is not concerned by what (s)he is going through.

There is a tradition of research studying the synchronization of EEGs and bold fMRI signals of two persons interacting, imitating each others’ movements [e.g., 33] and of persons going through the same stimulation(s) [e.g., 34, for a review, see 35]. This tradition could be relevant since, here, we also recorded the EEG of two participants simultaneously. However, we used ERPs, not EEGs’ synchrony or fMRI, and our participants were not interacting, imitating each other, or being presented with only the same stimulation. Each subject in a pair was going through the experiment on his/her own “despite” the fact that (s)he was sitting side by side with a friend/sibling/spouse. Sameness, and belief in that sameness, were manipulated, which both modulated the amplitude of a well-known ERP index of consciousness. To the best of our knowledge, there is thus yet no equivalent to the present study. The hypothesis of a direct sharing of qualia has never been tested. Future studies have to explore whether differential EEG synchrony can also occur within the present design and also test whether qualia sharing could account for part of the EEG synchrony observed in interacting participants. Indeed, the conscious intention to perform an action, when imitating, can be considered as a qualia and could, according to the present results, impact the functioning of the brain of the interacting person.

It has to be noted that, if further replicated, these findings could open several avenues of research. For instance, it might be interesting to explore whether young children’s brains, or recovering brain damaged patients, learn to produce their qualia with the help of others. It could also be interesting to see if autistic children suffer from a disability of this learning mechanism or whether their tendency to limit contact with others is a strategy that protects them against a deficit of the filtering mechanism.

In any case, the results of the present study provide preliminary data that can be used to support the existence of direct and spontaneous brain-to-brain communications. Further studies are necessary to replicate these findings, to determine the physical bases of these communications, to see whether or not they also occur between persons who are not closely related to each other and to establish if these communications are associated to the mechanisms by which qualia pertaining to the same stimulus could be similar across individuals, a similarity that is assumed in almost every social interaction.

## Data availability

The data referenced by this article are under copyright with the following copyright statement: Copyright: © 2018 Bouten S et al.

Data associated with the article are available under the terms of the Creative Commons Zero "No rights reserved" data waiver (CC0 1.0 Public domain dedication). 

F1000Research: Dataset 1. Mean voltages of the event-related brain potentials elicited by the IAPS stimuli in the P600 time window, 10.5256/f1000research.5977.d100682 ^36^

F1000Research: Dataset 2. Mean voltages of the event-related brain potentials elicited by the IAPS stimuli in the N400 time window, 10.5256/f1000research.5977.d100683 ^37^

F1000Research: Dataset 3. Mean voltages of the event-related brain potentials elicited by the IAPS stimuli in the P600 time window for BS and BD conditions, 10.5256/f1000research.5977.d100684 ^38^

## References

[1] Searle JR: How to study consciousness scientifically. *Philos Trans R Soc Lond B Biol Sci.* 1998;353(1377):1935–1942. 10.1098/rstb.1998.0346 9854266 PMC1692422

[2] Dennett DC: Quining Qualia. In *Consciousness in contemporary science* A J Marcel & E Bisiach Eds. Oxford Science publication, Clarendon Press Oxford.1988;43–77. Reference Source

[3] Petitot J Smith B: Physics and the phenomenal world. In R. Poli and P. M. Simons (eds.), *Formal Ontology*, Dondrecht/Boson/London: Kluwer.1997;223–254. Reference Source

[4] Ehrsson HH: The experimental induction of out-of-body experiences. *Science.* 2007;317(5841):1048. 10.1126/science.1142175 17717177

[5] Lakoff G Johnson M: Metaphors we live by. Chicago, Illinois, University of Chicago Press,1980 Reference Source

[6] Giora R: Understanding figurative and literal language: the graded salience hypothesis. *Cogn Linguist.* 1997;8(3):183–206. 10.1515/cogl.1997.8.3.183

[7] Feuillet L Dufour H Pelletier J: Brain of a white-collar worker. *Lancet.* 2007;370(9583):262. 10.1016/S0140-6736(07)61127-1 17658396

[8] Livingstone M Hubel D: Segregation of form, color, movement, and depth: anatomy, physiology, and perception. *Science.* 1988;240(4853):740–749. 10.1126/science.3283936 3283936

[9] Penfied W Jasper H: Epilepsy and the functional anatomy of the human brain. Litlle, Brown, Boston.1954;986 10.1093/brain/77.4.639

[10] Jones MW: Electromagnetic-Field Theories of the mind. *J Conscious Stud.* 2013;20:11–12. Reference Source

[11] Hameroff S Penrose R: Consciousness in the universe: a review of the ‘Orch OR’ belief. *Phys Life Rev.* 2014;11(1):39–78. 10.1016/j.plrev.2013.08.002 24070914

[12] Tressoldi P Pederzoli L Bilucaglia M: Brain-to-Brain (mind-to-mind) interaction at distance: a confirmatory study [v3; ref status: approved 1, not approved 1, http://f1000r.es/4ka]. *F1000Res.* 2014;3:182 10.12688/f1000research.4336.3

[13] Gratton G Bosco CM Kramer AF: Event-related brain potentials as indices of information extraction and response priming. *Electroencephalogr Clin Neurophysiol.* 1990;75(5):419–432. 10.1016/0013-4694(90)90087-Z 1692277

[14] Donchin E Coles MGH: Is the P300 a manifestation of context up-dating. *Behav Brain Sci.* 1988;11(3):357–374. 10.1017/S0140525X00058027

[15] Brower H Fitz H Hoeks J: Getting real about semantic illusion: rethinking the functional role of the P600 in language comprehension. *Brain Res.* 2012;1446:127–143. 10.1016/j.brainres.2012.01.055 22361114

[16] Lang PJ Bradley MM Cuthbert BN: International affective picture system (IAPS): Technical manual and affective ratings. The Center for Research in Psychophysiology, University of Florida, Gainesville, FL,1997 Reference Source

[17] Tecce JJ: Contingent negative variation (CNV) and psychological processes in man. *Psychol Bull.* 1972;77(2):73–108. 10.1037/h0032177 4621420

[18] Electrode position nomenclature committee: American Electroencephalographic Society guidelines for standard electrode position nomenclature. *J Clin Neurophysiol.* 1991;8(2):200–202. 10.1097/00004691-199104000-00007 2050819

[19] Geisser S Greenhouse GW: On methods in the analysis of profile data. *Psychometrika.* 1959;24(2):95–112. 10.1007/BF02289823

[20] Friedman D Trott C: An event-related potential study of encoding in young and older adults. *Neuropsychologia.* 2000;38(5):542–557. 10.1016/S0028-3932(99)00122-0 10689032

[21] Paller KA Kutas M Mayes AR: Neural correlates of encoding in an incidental learning paradigm. *Electroencephalogr Clin Neurophysiol.* 1987;67(4):360–371. 10.1016/0013-4694(87)90124-6 2441971

[22] Dumas G de Guzman GC Tognoli E: The human dynamic clamp as a paradigm for social interaction. *Proc Natl Acad Sci U S A.* 2014;111(35):E3726–34. 10.1073/pnas.1407486111 25114256 PMC4156776

[23] Van Gaal S Naccache L Meuwese JD: Can the meaning of multiple words be integrated unconsciously? *Philos Trans R Soc Lond B Biol Sci.* 2014;369(1641):20130212. 10.1098/rstb.2013.0212 24639583 PMC3965166

[24] Vogel EK Luck SJ Shapiro KL: Electrophysiological evidence for a postperceptual locus of suppression during the attentional blink. *J Exp Psychol Hum Percept Perform.* 1998;24(6):1656–1674. 10.1037/0096-1523.24.6.1656 9861716

[25] Dobson J Grassi P: Magnetic properties of human hippocampal tissue--evaluation of artefact and contamination sources. *Brain Res Bull.* 1996;39(4):255–259. 10.1016/0361-9230(95)02132-9 8963692

[26] Dunn JR Fuller M Zoeger J: Magnetic material in the human hippocampus. *Brain Res Bull.* 1995;36(2):149–153. 10.1016/0361-9230(94)00182-Z 7895092

[27] Kirschvink JL Kobayashi-Korschvink A Woodford BJ: Magnetite biomineralization in the human brain. *Proc Natl Acad Sci U S A.* 1992;89(16):7683–7687. 10.1073/pnas.89.16.7683 1502184 PMC49775

[28] Solov’yov IA Schulten K: Reaction kinetics and mechanism of magnetic field effects in cryptochrome. *J Phys Chem B.* 2012;116(3):1089–99. 10.1021/jp209508y 22171949 PMC3266978

[29] Burda H Begall S Cervený J: Extremely low-frequency electromagnetic fields disrupt magnetic alignment of ruminants. *Proc Natl Acad Sci U S A.* 2009;106(14):5708–5713. 10.1073/pnas.0811194106 19299504 PMC2667019

[30] Hart V Nováková P Malkemper EP: Dogs are sensitive to small variations of the Earth’s magnetic field. *Front Zool.* 2013;10(1):80. 10.1186/1742-9994-10-80 24370002 PMC3882779

[31] Persingerr MA Lavalléer CF: Theoretical and Experimental Evidence of Macroscopic Entanglement Between Human Brain Activity and Photon Emissions: Implications for Quantum Consciousness and Future Applications. *J Consciousness Explor Res.* 2010;1(7):785–807. Reference Source

[32] Weidenmüller M: Quantum physics: spooky action gets collective. *Nature.* 2013;498(7455):438–439. 10.1038/nature12259 23783516

[33] Burgess AP: On the interpretation of synchronization in EEG hyperscanning studies: a cautionary note. *Front Hum Neurosci.* 2013;7:881. 10.3389/fnhum.2013.00881 24399948 PMC3870947

[34] Hasson U Nir Y Levy I: Intersubject synchronization of cortical activity during natural vision. *Science.* 2004;303(5664):1634–40. 10.1126/science.1089506 15016991

[35] Hasson U Ghazanfar AA Galantucci B: Brain-to-brain coupling: a mechanism for creating and sharing a social world. *Trends Cogn Sci.* 2012;16(2):114–121. 10.1016/j.tics.2011.12.007 22221820 PMC3269540

[36] Boutenr S Debruiller JB: Dataset 1 in: Finding spontaneous brain-to-brain communications when looking for a cause of the similarity of qualia assumed across individuals. *F1000Research.* 2015 Data Source

[37] Boutenr S Debruiller JB: Dataset 2 in: Finding spontaneous brain-to-brain communications when looking for a cause of the similarity of qualia assumed across individuals. *F1000Research.* 2015 Data Source

[38] Boutenr S Debruiller JB: Dataset 3 in: Finding spontaneous brain-to-brain communications when looking for a cause of the similarity of qualia assumed across individuals. *F1000Research.* 2015 Data Source

