# Supplementary material for: Looking for effects of qualia on event-related brain potentials of close others in search for a cause of the similarity of qualia assumed across individuals
Source: F1000Res. 2018 Jun 4;3:316. Originally published 2014 Dec 29. [Version 3] doi: 10.12688/f1000research.5977.3 (PMC5992535; doi:10.12688/f1000research.5977.3)
Supplement: Peer review history

# Peer review: Referee response for version 3

**DOI:** 10.5256/f1000research.16512.r34659

**Published:** 2018-06-06

**Authors:** Claude Messier

**Affiliations:** 1 School of Psychology, University of Ottawa, Ottawa, ON, Canada

I have no further comments

I have read this submission. I believe that I have an appropriate level of expertise to confirm that it is of an acceptable scientific standard.

# Peer review: Referee response for version 2

**DOI:** 10.5256/f1000research.7505.r32859

**Published:** 2018-05-04

**Authors:** Claude Messier

**Affiliations:** 1 School of Psychology, University of Ottawa, Ottawa, ON, Canada

“Qualia” are always an interesting topic because it appears to bridge philosophy, psychology, physiology and our own experience in a way that very little can be argued beyond beliefs. Two questions are raised by this communication. The first one is whether humans share the same qualia and the second is whether an individual’s qualia can be influenced by the qualia of another individual.

 The first question has been examined by many experiments in vision research. One way to answer this question is to find exceptions to the shared qualia. For vision, an obvious example is the variation of opsin genes in retinal cones (OPN1SW OPN1MW or OPN1LW). There is also the contested possibility (Jameson et al,  2014; 2016) that a small proportion of women have 4 opsin genes (tetrachromat) – an occurrence that is common in many animals (birds, frogs, insects Scholtyßek et al, 2017). Qualia are clearly affected by the different inputs from the retina containing variants of the opsin distribution in humans. Developmental color blindness is usually based on these variants.

 There is also acquired color blindness of toxic origin in the retina (Choi, 2017) or from selective cortical lesions that induce achromatopsia within a visual field with preserved vision (Beauchamp et al. 2000). Self-reports in case study of acquired cortical achromatopsia suggest that all qualia modes (conscious perception, imagery and dreams) are equally affected (Sacks, 1987). Finally, the most common manifestation of synesthesia involves color percept on black and white letters.

 Color synesthesia either acquired or developmental is associated with unusual connectivity between various brain areas and the “V4” area in the visual cortex (Amsel, 2017). Developmental synesthesia has a genetic component that is being examined (Tilot et al., 2018).

 These “abnormal” acquired variants and rare developmental variants are relatively clear demonstration that qualia, in this case color qualia, can differ among individuals. Many people with developmental color-word synesthesia are often unaware that their color qualia is different until they are older suggesting that many subtle variants are unknown because people are unaware that they are at variant with the general population. Several factors also appear to influence color perception and cognitive identification including aging, as well as language and culture

 I now return to the majority of humans that have the expected cones and opsins as well as brain connectivity that apparently produce typical color perception and we assume typical qualia. Do these prerequisites of color perception sufficient to produce a common color qualia in the majority of humans. Although the source of variation is enigmatic, two people can have very different sensitivities to wavelength and label the same physical stimuli very differently (Webster, 2014). The most important source of variation comes from the differences in macular and lens pigment density (Webster & MacLeod, 1988). A common polymorphism in the OPN1LW gene produce a small but functional shift in the peak spectral sensitivity for long-wave light (Winderickx et al, 1992). Finally there is a large variation in the relative number of each of the three cones across people with normal vision (Hofer, Carroll, Neitz, Neitz, & Williams, 2005).

 This lengthy introduction to this review suggest that humans, in general, do live in a delusional world where they think that all people experience the basic aspects of colour perception more or less exactly as they do. This delusion is mostly hidden behind our other delusion that even though colors are perceived the same, people can have preferred colors and can evaluate coloured garments as matching differently. However we know that social pressures can lead to temporary social consensus regarding the aesthetics of certain colours (e.g. the “avocado green” and “harvest gold” appliances of the 1970’s).

 The present experiment examines the impact of the knowledge of another person perceptual judgement can influence or bias another person. In order to remove qualitative estimates, the experimenter chose to measure evoked potentials. Two participants were tested at the same time and presented simultaneously (but out of view of the other participant) two pictures that could be either the same or two different pictures. Before each trial, each participant was told whether the other participant was viewing a pair of pictures that were the same or different.

 The question asked was whether knowledge of what the other participant was seeing (same/different) was sufficient to induce different evoked potentials patterns in the 4 conditions. The authors found that in the inconsistent conditions induced a slight variation in the P600 evoked potential in all electrode groups (e.g. the pictures presented were the same but the participant was told that the other person would see different pictures in their pair). The key event here is that the participants were misled about the pictures seen by the other participant – either by displaying a statement saying that the pictures would be different but were not OR that picture would be the same but were actually different. These two conditions were contrasted with two other conditions where the statements were congruent with the presentations. The authors argue that because the participants had no way of overtly knowing whether the statements were true or not, that information had to be exchanged directly from brain to brain.

 Although I understand the enthusiasm of the authors regarding this possibility, one has to realize that we do exchange information between brains using numerous sensory/perceptual channels – some of which are obvious (like language) and some less obvious. In the experiments, the authors did take pains to verify some of the obvious channels that would not rely on “brain-to-brain” information exchange. However because this type of exchange is considered very unlikely by most, the authors should be held to a much higher standard to eliminate all other possibilities – some of which could be very subtle.

 Although I have not experienced the testing situation, I can only offer some speculations. For example, it is likely that the luminance of the pictures presented on the two halves of the screen shared by participants resulted in some reflected glare that could be somehow be detected – possibly without consciousness. Also, the screen could present subtle differences when presenting two identical pictures compared to different pictures on the two half-screen portions. The point here is that exceptional claims have to be tested in several experimental contexts that are highly controlled before one allows the support of unconventional hypotheses. This is a typical human and scientific bias but not an unreasonable one. Sometimes this higher standard of proof slows down science progress but sometimes it uncovers uncontrolled variables that refute the unconventional hypothesis. An example of an additional test of the brain-to-brain hypothesis would be to use auditory information presented with soundproof earphones.

 As it stands, we have interesting results and the authors propose an unconventional hypothesis based on the absence, for them, of other alternative hypotheses. As the saying goes, additional data are needed.

I have read this submission. I believe that I have an appropriate level of expertise to confirm that it is of an acceptable scientific standard, however I have significant reservations, as outlined above.

## References

[1] HoferHCarrollJNeitzJNeitzMWilliamsDR: Organization of the human trichromatic cone mosaic. *J Neurosci*.2005;25(42) : 10.1523/JNEUROSCI.2414-05.2005 9669-79 10.1523/JNEUROSCI.2414-05.2005 16237171PMC6725723

[2] JamesonK.A.WinklerA.D.HerreraC.GoldfarbK.: The Veridicality of Color: A case study of potential human tetrachromacy. *Technical Report Series MBS 14-02. Institute for Mathematical Behavioral Sciences University of California at Irvine. Irvine, CA, USA*.2014; Reference source

[3] JamesonK.A.WinklerA.D.GoldfarbK.: Art, interpersonal comparisons of color experience, and potential tetrachromacy. Invited Proceedings paper for the 2016 ISandT International Symposium on Electronic Imaging (EI 2016). *Technical Session on Human Vision and Electronic Imaging*.2016;1-12

[4] ScholtyßekCKelberA: [Color vision in animals : From color blind seals to tetrachromatic vision in birds]. *Ophthalmologe*.2017;114(11) : 10.1007/s00347-017-0543-6 978-985 10.1007/s00347-017-0543-6 28752388

[5] ChoiARBraunJMPapandonatosGDGreenbergPB: Occupational styrene exposure and acquired dyschromatopsia: A systematic review and meta-analysis. *Am J Ind Med*.2017;60(11) : 10.1002/ajim.22766 930-946 10.1002/ajim.22766 28836685PMC5652067

[6] BeauchampMSHaxbyJVRosenACDeYoeEA: A functional MRI case study of acquired cerebral dyschromatopsia. *Neuropsychologia*.2000;38(8) :1170-9 1083815110.1016/s0028-3932(00)00017-8

[7] TilotAKKuceraKSVinoAAsherJEBaron-CohenSFisherSE: Rare variants in axonogenesis genes connect three families with sound-color synesthesia. *Proc Natl Acad Sci U S A*.2018;115(12) : 10.1073/pnas.1715492115 3168-3173 10.1073/pnas.1715492115 29507195PMC5866556

[8] AmselBDKutasMCoulsonS: Projectors, associators, visual imagery, and the time course of visual processing in grapheme-color synesthesia. *Cogn Neurosci*.2017;8(4) : 10.1080/17588928.2017.1353492 206-223 10.1080/17588928.2017.1353492 28697672PMC6559728

[9] ElliotA.J.: Individual Differences in Color Vision Michael A. Webster Handbook of Color Psychology AJ Elliot, MD Fairchild, and A. Franklin, Eds. *Cambridge University Press*.2015;197-215

[10] WebsterMAMacLeodDI: Factors underlying individual differences in the color matches of normal observers. *J Opt Soc Am A*.1988;5(10) :1722-35 320443510.1364/josaa.5.001722

[11] WinderickxJLindseyDTSanockiETellerDYMotulskyAGDeebSS: Polymorphism in red photopigment underlies variation in colour matching. *Nature*.1992;356(6368) : 10.1038/356431a0 431-3 10.1038/356431a0 1557123

# Response

**Published:** 2018-05-30

**Authors:** J. Bruno Debruille

**Affiliations:** McGill University, Canada

Thank you for this very interesting review.  We learned quite a few things about the variations of color perceptions and do accept that some individuals, due to their biology, have color qualia that differ from those of others. We also agree with the conclusion that:

 "humans, in general, do live in a delusional world where they think that *all* people experience the basic aspects of color perception more or less *exactly* as they do." because of the words we italicized in that quote. In effect, these words allow us to maintain our claim that, *globally*, a vast number of people experience color qualia in a *similar* way.

 It is then logical to ask how that can be whereas this similarity does not appear to be based on neural networks' similarity. In effect, after a stroke damaging the visual cortex, some patients recover the ability to generate old qualia in new areas and thus in other neural networks. Or, in blind people, it seems that the visual cortex can generate sound qualia.

 One simple and straightforward way that a vast number of people can experience similar qualia is by influencing each other. This way, the brains of children, for instance, which are both macroscopically and microscopically different from those of adults, can learn to generate qualia similar to those of their parents. We thus maintain that there is a logic in looking for an impact of qualia on the brain of others.

 We acknowledge that hypothesizing such an impact is assuming that qualia have a physical nature (which then remain to be discovered). But, would it be scientific to talk about something without assuming such a nature? Wouldn’t that create the mind-body problem by saying that brains, which are material, could create something (i.e., qualia) that have no physical nature?

 For many, qualia *emerge* from particular patterns of neural activation and those patterns are all the physics involved. However, this emergence idea contrasts with the 3 D movie (Chalmers), which is made by the brain integrating the various qualia it created into a perceived world in which we feel. How could one say that this movie *is* such patterns? It appears very counterintuitive. To illustrate this counter intuitiveness, one could transpose the emergence idea to computers. One would then be saying that the activity of the graphic processing unit, for instance, would *be* the visual display, which does not make sense. Moreover, restricting each quale to the particular pattern of neural activity, from which it would emerge, leaves us with problem created by the possible lack of neural networks’ similarities mentioned above.

 So, it makes sense to say that the qualia generated by the brain a) have their own physics and b) can be generated by different neural networks provided that these networks learn to develop the appropriate activity.

 Saying that qualia are physical makes it easier to understand why our brains are sensitive to them, as since shown by our reactions to the perceived world.

 And, if brains are sensitive to the qualia they created, there is an easy way to understand how a vast number of people can learn to produce similar qualia: again, by a sensitivity of their brains to the qualia of others.

 We want to emphasize that logic because, to the best of our knowledge, it is the first time that it is used.

 Response to comment two:

 “ In the experiments, the authors did take pains to verify some of the obvious channels that would not rely on “brain-to-brain” information exchange. However because this type of exchange is considered very unlikely by most, the authors should be held to a much higher standard to eliminate all other possibilities – some of which could be very subtle. »

 « Although I have not experienced the testing situation, I can only offer some speculations. For example, it is likely that the luminance of the pictures presented on the two halves of the screen shared by participants resulted in some reflected glare that could be somehow be detected – possibly without consciousness. Also, the screen could present subtle differences when presenting two identical pictures compared to different pictures on the two half-screen portions. »

 We agree. This glare is indeed something we should have thought about.

 We thus went back in the testing room and run the stimulus sequence.

 Just as when participants were there during the experiment, we switched on the dim lights (=two alarm bulbs powered by a battery) that are on the ceiling of the participants’ room.

 During the display of the stimuli, the walls of that room appear to reflect only the light of the dim lights, not the light from the images appearing on the computer screen, which is too weak. We also look at the screen itself to see whether the white that was surrounding IAPS images depends a bit on the image presented on the other half of the screen. None of the members of the lab present at that time were able to detect changes in that white along with the display of the images, leave alone using that to say whether the two images were the same or different. The white screen surrounding the image just does not appear to change when images occur.

 On the other hand, in a follow-up study, we used the faces of the MED-bank, which are photographs of people who were shot on the same background, using the same lighting, lens and film. These pictures thus induce similar glares. Nevertheless, we still found an effect of qualia on ERPs of close others with those other stimuli.

 On the other hand, in a follow-up paper that has just been submitted at BioRxiv, we separated the two partners of each pair with a black curtain, which went much higher than their heads. This was done so that they could not see each other in the very periphery of their visual field. Nevertheless, the curtain also prevented them from seeing the wall that was next to the half of the screen of the partner, and thus from detecting a glare of the image presented to this partner. In this setting, we also found an effect of qualia on the ERPs of 32 close-others.

 In this follow-up paper, we also had a group of 34 strangers. These latter participants, who never saw each other before the experiment, did not have significant effect of qualia of others on their ERPs, despite the fact that they were not separated by a curtain.

 We would like also to point again the particular nature of the ERP effects obtained on the N400 and on the late posterior-central positivity (i.e., the LPP, also named the late positive complex or the P600). The fact that the N400 was significantly smaller for stimuli which were *in*consistent than for those which were consistent and that LPPs were greater is not something we could have had by *presenting* incompatible information to participants. When such information occurs and is consciously detected by participants in usual designs, N400s are well-known to be larger and P600s smaller.

 To conclude, we would say that there is thus more than one reason for which it is very difficult to account for the present ERP effects using “classical” human to human communications. Nevertheless, we are of course running other studies to replicate and extend.

# Peer review: Referee response for version 2

**DOI:** 10.5256/f1000research.7505.r10137

**Published:** 2015-10-29

**Authors:** André Achim

**Affiliations:** 1 Department of Psychology, Université du Québec à Montréal, Montréal, QC, Canada

The new title usefully resets the scope of the present work. Although the origin of the project remains the hunt for an electrophysiological correlate of qualia and the discussion retains such aspect, the present version of the research report settles on a more modest, but still quite bold, interpretation of the ERP results. Hopefully, the readers of the report will acknowledge the data and their statistical analyses, and will take the authors’ speculations about qualia and about direct brain-to-brain communication as possible although not necessary explanations. Since direct brain-to-brain communication is quite close to parapsychology, and especially to extra-sensory perception (ESP), the topic calls for an extra amount of skepticism.

It is unfortunate that the authors chose to move the data analysis window from 600-900 ms in the first version to 650-950 ms post stimulus onset in the present version, although not consistently so since the effect of belief still uses the 600-900 ms range. The change in analysis interval seems related to explorations aimed at improving the statistical significance of the data, which would be a questionable scientific practice. I will therefore assume that the *a priori *specification of the interval of interest, presumably not guided by inspection of the data, is the 600-900 ms range.

In the present version of the report, the authors substituted two separate ANOVAs to their original Sameness x Belief 2X2 design. In the present version, the analysis for the Belief effect, also referred to as the social cognition effect, remains the same as in the 2x2 design, and the Concordance effect, except for the 50 ms shift in the data interval, is the same as the interaction in the previous 2x2 design. Had the analysis remained 2x2 but with Concordance as a main effect, then the Concordance x Belief interaction would have been the Sameness main effect of the earlier 2x2 analysis. Sticking to the *a priori* selected 600-900 ms interval allows discussing the interaction that is missing in the present revision. The authors’ explanation that averaging over two conditions for each of the separate ANOVAs (e.g. over the two Belief conditions for each Concordance condition for the ANOVA on the Concordance effect) should improve the signal-to-noise ratio is an oversight (except for the graphs) for statistical analysis since the 2x2 ANOVA already achieved that effect (averaging the two amplitude values of each subject is equivalent to computing the amplitude of the common averaged waveform, with very mild modulation by unequal number of stimuli in the two conditions pooled together).

The reasoning leading from considerations of qualia to the analysis of ERP amplitude in the time interval of the P600 appears extremely opaque. If, for instance, the specific subjective experience of blue in one person was to be shared through the modulation of EEG signals, this modulation would need to differ from that of the subjective experience of green. Since each qualia would need its own signature, measuring the P600 amplitude can hardly reflect the information. With the shift in emphasis from qualia to direct brain-to-brain communication, similar problems remain undiscussed: What would be the contents of that direct brain-to-brain communication? Is the effect rich enough to convey a reasonably large variety of contents?

The possibility that the ERP effects index direct brain-to-brain communication is definitely worth reporting. Before accepting the conclusion as quite likely, however, the readers should await elimination of alternate, more mundane, explanations. In the present revision, the authors acknowledge the possibility that subtle cues would be transmitted from one participant to the other in reaction to some emotional stimuli, but argue that the time required for one participant’s brain to process the information and emit a bodily response plus the time required by the partner to perceive and process that response is inconsistent with the time of onset of the observed ERP difference. There are other possibilities however that will need to be ruled out experimentally for the direct brain-to-brain interpretation to hold. The subconscious perception, in the DBs condition, that the partner reacts emotionally (perhaps expressed by a contraction of the hand) when the currently seen picture does not justify it could alert the participant for extra processing of the sequence of stimuli. This, however, does not have to be a strict stimulus by stimulus effect and could well affect the processing of upcoming stimuli. For the SBd condition, the other member of the non-concordant condition, this alternate explanation is, however, not so obvious to formulate.

The lack of condition differences among SBs, SBd and DBs, reported in my comments on the previous version does not support the alternate interpretation formulated here, although only SBs is expected to differ from the other two in the context of a concordance effect. To further question the merit of the alternate hypothesis, there is interest in the interaction between the two dimensions analyzed in the present version. This is the Sameness effect in the earlier report, which was significant or marginally so in the parasagittal (p=.07) and lateral (p=.04) electrode sets. It is thus conceivable that the Concordance effect and its interaction with Belief would indicate that the Concordance effect is more important (perhaps only present) for one of the four test conditions. It becomes relevant to assess whether the interaction suggests that the concordance effect would indeed be stronger for DBs compared to SBs than for DBd compared to SDd. If the effect was essentially that of DBs differing from the remaining three conditions, a Belief effect should also be expected. No Belief effect was reported in Table 2 of the previous version, but Table 3 documents significant differences of DBd with both of DBs (a Belief effect) and SBd (a Sameness effect). How would the concordance effect be re-interpreted if it was present only for the believe-different conditions (seen in Table 3 of previous version) but not for the believe-same conditions (i.e. SBs vs DBs)?

Other forms of alternate interpretations remain possible. For instance, the SBd condition could induce the subconscious perception that the partner is moved similar to oneself, leading to the suspicion that the stimuli, although different, would be matched for emotional contents. This could also add the extra processing of the remaining stimuli in the block. The statement in the abstract that the participants had no way to detect inconsistence is premature since it relies essentially on each participant not seeing the stimulus presented to the partner. I agree however that there was no obvious way by which the information about inconsistency (or perhaps about consistency) could be conveyed by visual, auditory or tactile cues; direct brain-to-brain communication remains a possibility, although one requiring the exclusion of simpler alternatives.

Later experimentations should therefore concentrate on eliminating the possibility that response cues (of perceptual-emotional origin) modulate the processing of later stimuli, by adding the dimension that not only the stimuli but also the partner’s reactions are monitored in some of the conditions. The direct brain-to-brain hypothesis does not require that the partners should see or could hear noises from each other, although it may be speculated that being aware of the presence of the other person would be required. Fitting the participants with ear plugs and with eye screens preventing lateral visual perception should retain the impression of the presence of the other while blocking most signs of emotional reaction (floor vibration would not be excluded).

Given the structure of publication in F1000Research, it would not be required that the authors amend the current version for the readers to become aware that simpler alternate explanations must be excluded before accepting the ESP interpretation. This being said, a number of more minor points are worth addressing.

**Contents:**

The possibility of lateral eye movements toward the partner is documented in the revised version by plotting the ERPs separately for participants on the left and on the right. By visual superposition of the graphs for F7 and F8 in Figure 5 A and B, there are hints for more positivity (in both conditions painted) on the side of the partner, which is the expected pattern of eye movement contamination, considering that the cornea is positive relative to the retina.

Page 4, column 2, “The 32 subjects of the 16 pairs underwent …” I find the simplification hardly acceptable, since the earlier version of the report and the accompanying data file provide evidence that there were only 14 intact pairs (13 d.f. in the correlations tests reported in my earlier comment). It would have been far better to describe how many dyads were tested and to explain on what grounds subjects were excluded.

**Opinion:**

In the abstract, "no way to see the stimulus their partner was presented with, and thus no way to detect inconsistence"’ is currently an over optimistic statement.

In the "Amendments from version 1" box, the authors mention averaging the consistent and the inconsistent conditions over the two believe conditions, partly to increase signal-to-noise. This is not at all an argument for not analysing belief and consistency effects in the same ANOVA, since the amplitude of an average is the same as the average of the amplitudes when there is an equal number of individual sweeps in the two conditions (and very nearly so for comparatively large numbers of sweeps).

**Clerical:**

In the abstract the word ‘inconsistent’ is split between two lines without hyphenation.

Page 4, column 2, line 8: "if, and only if, that there is no way"

In the Data Acquisition paragraph, only 26 of the 28 channels are listed, FP1 and FP2 present in the figures are missing here.

Page 11, 2nd paragraph, “if” is missing in “Indeed, this were the case, one would have …”.

Page 14, first paragraph, “of” is missing in “to verify the absence errors”.

I have read this submission. I believe that I have an appropriate level of expertise to confirm that it is of an acceptable scientific standard.

# Response

**Published:** 2016-09-05

**Authors:** J. Bruno Debruille

**Affiliations:** McGill University, Canada

The new title usefully resets the scope of the present work. Although the origin of the project remains the hunt for an electrophysiological correlate of qualia and the discussion retains such aspect, the present version of the research report settles on a more modest, but still quite bold, interpretation of the ERP results. Hopefully, the readers of the report will acknowledge the data and their statistical analyses, and will take the authors’ speculations about qualia and about direct brain-to-brain communication as possible although not necessary explanations. Since direct brain-to-brain communication is quite close to parapsychology, and especially to extra-sensory perception (ESP), the topic calls for an extra amount of skepticism.

**We agree with these comments for the most part. However, parapsychology and telepathy do not focus on the means by which our percepts of a real stimulus can be similar to the percepts generated by that stimulus in other individuals despite brain differences. Moreover, the word parapsychology suggests an explicit quest for facts and data that would be somehow apart from psychology. On the contrary, our goal is to account for a usual psychology fact.**

 It is unfortunate that the authors chose to move the data analysis window from 600-900 ms in the first version to 650-950 ms post stimulus onset in the present version, although not consistently so since the effect of belief still uses the 600-900 ms range. The change in analysis interval seems related to explorations aimed at improving the statistical significance of the data, which would be a questionable scientific practice. I will therefore assume that the *a priori*specification of the interval of interest, presumably not guided by inspection of the data, is the 600-900 ms range.

**The time window of measure has not been moved, neither for consistency nor for belief! Exploring the latency of early ERPs, we detected they appeared to peak too early. We run tests. They proved that the photo-transistor placed on the screen to detect the real time of onset of the stimuli was providing the EEG computer with a trigger that was systematically delayed by 50 ms. In the second version of the paper, we decided to take that into account. So, we were already measuring the mean voltage between 650 to 950 ms in the first version. In the second version, we thus mentioned this real time window of measure and corrected Figures 2 and 3, which thus now include the -.16 s. in the x axis.**

 In the present version of the report, the authors substituted two separate ANOVAs to their original Sameness x Belief 2X2 design. In the present version, the analysis for the Belief effect, also referred to as the social cognition effect, remains the same as in the 2x2 design, and the Concordance effect, except for the 50 ms shift in the data interval, is the same as the interaction in the previous 2x2 design. Had the analysis remained 2x2 but with Concordance as a main effect, then the Concordance x Belief interaction would have been the Sameness main effect of the earlier 2x2 analysis. Sticking to the *a priori* selected 600-900 ms interval allows discussing the interaction that is missing in the present revision. The authors’ explanation that averaging over two conditions for each of the separate ANOVAs (e.g. over the two Belief conditions for each Concordance condition for the ANOVA on the Concordance effect) should improve the signal-to-noise ratio is an oversight (except for the graphs) for statistical analysis since the 2x2 ANOVA already achieved that effect (averaging the two amplitude values of each subject is equivalent to computing the amplitude of the common averaged waveform, with very mild modulation by unequal number of stimuli in the two conditions pooled together).

**Yes, the interaction between belief and reality (=sameness) is equivalent to the concordance effect. But we think it is easier to understand this concordance than to speak about an interaction between these two factors. Moreover, taking advantage of the format of this journal, readers will have both approach by looking at the first and second version. For the 600-900 to 650-950, please see our response to the previous comment.**

 The reasoning leading from considerations of qualia to the analysis of ERP amplitude in the time interval of the P600 appears extremely opaque. If, for instance, the specific subjective experience of blue in one person was to be shared through the modulation of EEG signals, this modulation would need to differ from that of the subjective experience of green. Since each qualia would need its own signature, measuring the P600 amplitude can hardly reflect the information. With the shift in emphasis from qualia to direct brain-to-brain communication, similar problems remain undiscussed: What would be the contents of that direct brain-to-brain communication? Is the effect rich enough to convey a reasonably large variety of contents?

**Thoughtful comment, and of critical importance. All we know is that (1) there is a relation between the amplitude of the late posterior positivity (LPP or P600) and putting new information into the “working memory part of consciousness” (see S. Luck and S. Dehaene work on attentional blink for instance). On the other hand, (2) the results of the present paper suggest that there could be direct brain to brain communications. Finally, (3) we know that such communications would be very useful to understand how qualia can be similar across individuals. The problem is how to link those these three elements. Many possibilities can be discussed. The most parsimonious hypothesis is that the brain activities responsible for the consciousness of a qualia are those that are responsible for a signal to which the brain of the partner is sensitive to. However, there is no way to know whether the emission of this signal and/ or its “reception” is indexed by the LPP. The modulation of this ERP could be an (a posteriori) consequence of the processing of that signal, which could be emitted during preconscious processing, rather than during the consciousness of the qualia….**

****

**This is now included in the discussion section of the version 3 of the paper, during the paragraph starting with “However…”** ***p 23.***

 The possibility that the ERP effects index direct brain-to-brain communication is definitely worth reporting. Before accepting the conclusion as quite likely, however, the readers should await elimination of alternate, more mundane, explanations. In the present revision, the authors acknowledge the possibility that subtle cues would be transmitted from one participant to the other in reaction to some emotional stimuli, but argue that the time required for one participant’s brain to process the information and emit a bodily response plus the time required by the partner to perceive and process that response is inconsistent with the time of onset of the observed ERP difference. There are other possibilities however that will need to be ruled out experimentally for the direct brain-to-brain interpretation to hold. The subconscious perception, in the DBs condition, that the partner reacts emotionally (perhaps expressed by a contraction of the hand) when the currently seen picture does not justify it could alert the participant for extra processing of the sequence of stimuli. This, however, does not have to be a strict stimulus by stimulus effect and could well affect the processing of upcoming stimuli. For the SBd condition, the other member of the non-concordant condition, this alternate explanation is, however, not so obvious to formulate.

**We agree that there was an unlikely possibility that a subconscious perception of such an unjustified alert could have impacted the processing of upcoming stimuli. This was thus added to the paper, end of p22, just before mentioning:  the impact of subliminal information on the processing of upcoming stimuli is usually very short. For instance, the priming induced by the prior presentation of a subliminal word that is semantically associated to an upcoming target words, lasts at most a few hundred milliseconds. Negative priming lasts longer but at most 8 seconds, which would only impact the two trials in the current experiment, and it depends on an effortful ignorance of distractors that may have been consciously perceived at first.**

 The lack of condition differences among SBs, SBd and DBs, reported in my comments on the previous version does not support the alternate interpretation formulated here, although only SBs is expected to differ from the other two in the context of a concordance effect. To further question the merit of the alternate hypothesis, there is interest in the interaction between the two dimensions analyzed in the present version. This is the Sameness effect in the earlier report, which was significant or marginally so in the parasagittal (p=.07) and lateral (p=.04) electrode sets. It is thus conceivable that the Concordance effect and its interaction with Belief would indicate that the Concordance effect is more important (perhaps only present) for one of the four test conditions. It becomes relevant to assess whether the interaction suggests that the concordance effect would indeed be stronger for DBs compared to SBs than for DBd compared to SDd. If the effect was essentially that of DBs differing from the remaining three conditions, a Belief effect should also be expected. No Belief effect was reported in Table 2 of the previous version, but Table 3 documents significant differences of DBd with both of DBs (a Belief effect) and SBd (a Sameness effect). How would the concordance effect be re-interpreted if it was present only for the believe-different conditions (seen in Table 3 of previous version) but not for the believe-same conditions (i.e. SBs vs DBs)?

**This is an interesting question. In the first version, we discussed some possibilities. It may be the case that, in young adults, and thus in individuals who have already learned to produce most qualia, the belief that their partner is seeing the same stimulus prevents their system from using the information, taking for granted that it already has everything. If this hypothesis were correct, there should then be a difference between the two believe-same conditions only in the case of children. Then, at some age, brains would be mainly "interested" in " trying to guess" what close ones go through in the case it differs from what we go through.**

 Other forms of alternate interpretations remain possible. For instance, the SBd condition could induce the subconscious perception that the partner is moved similar to oneself, leading to the suspicion that the stimuli, although different, would be matched for emotional contents. This could also add the extra processing of the remaining stimuli in the block. The statement in the abstract that the participants had no way to detect inconsistence is premature since it relies essentially on each participant not seeing the stimulus presented to the partner. I agree however that there was no obvious way by which the information about inconsistency (or perhaps about consistency) could be conveyed by visual, auditory or tactile cues; direct brain-to-brain communication remains a possibility, although one requiring the exclusion of simpler alternatives.

**Yes. We agree in principle. Although we think it is very unlikely that a  “subconscious perception that the partner is moved similar to oneself, leads to the ’unconscious’ (our addition) suspicion that the stimuli, although different, is matched for emotional contents.” See also above, in response to a prior comment, the duration of the effects of subliminal information.**

 Later experimentations should therefore concentrate on eliminating the possibility that response cues (of perceptual-emotional origin) modulate the processing of later stimuli, by adding the dimension that not only the stimuli but also the partner’s reactions are monitored in some of the conditions. The direct brain-to-brain hypothesis does not require that the partners should see or could hear noises from each other, although it may be speculated that being aware of the presence of the other person would be required. Fitting the participants with ear plugs and with eye screens preventing lateral visual perception should retain the impression of the presence of the other while blocking most signs of emotional reaction (floor vibration would not be excluded).

****

**Good ideas. Thank you. We have run a study with a black curtain separating the partners. The consistency effects remained (submitted). For next studies, we are also considering making a video of the participants during the experiment to check their movements and the noise they could make.**

 Given the structure of publication in F1000Research, it would not be required that the authors amend the current version for the readers to become aware that simpler alternate explanations must be excluded before accepting the ESP interpretation. This being said, a number of more minor points are worth addressing.

**Contents:**

 The possibility of lateral eye movements toward the partner is documented in the revised version by plotting the ERPs separately for participants on the left and on the right. By visual superposition of the graphs for F7 and F8 in Figure 5 A and B, there are hints for more positivity (in both conditions painted) on the side of the partner, which is the expected pattern of eye movement contamination, considering that the cornea is positive relative to the retina.

**We agree that F7 ERPs for participants who were on the right of their partners look smaller than ERPs of participants who were on the left of their partner. This could indicate differential eye movements. However, we have been deceived many times by comparing ERPs across subjects with less than 20 participants per group, and here, we only have 16 for each of these subgroups. Moreover, notable eye movements trigger huge effects on waves. Thus, even if the F7/ F8 differences signaled were reliable, it would indicate, at most, subtle eye movements, thus totally out of proportion to what would be necessary to focus on the other participant and his/her reactions. And, as mentioned, even looking side ways could not help them to see the stimulus presented to their partners. For this, they would also have had to move their body or to move the cardboard.**

****

 Page 4, column 2, “The 32 subjects of the 16 pairs underwent …” I find the simplification hardly acceptable, since the earlier version of the report and the accompanying data file provide evidence that there were only 14 intact pairs (13 d.f. in the correlations tests reported in my earlier comment). It would have been far better to describe how many dyads were tested and to explain on what grounds subjects were excluded.

**Four subjects of the initial 16 pairs were excluded because of problematic ERPs. For them, too many trials had to be rejected and hardly any ERPs could be obtained, a frequent problem when having no reaction time task. These 4 subjects did not belong to the same pairs. There were only 12 intact pairs. Two more pairs were thus recruited to reach 32 participants, which is why there was only 14 intact pairs at the end.**

****

**The Participants paragraph of the Method section was corrected accordingly** ***(p 8).***

**Opinion:**

 In the abstract, "no way to see the stimulus their partner was presented with, and thus no way to detect inconsistence"’ is currently an over optimistic statement. 

**Agreed. we added « a priori » (and thus: “a priori no way to detect inconsistence”).**

 In the "Amendments from version 1" box, the authors mention averaging the consistent and the inconsistent conditions over the two believe conditions, partly to increase signal-to-noise. This is not at all an argument for not analysing belief and consistency effects in the same ANOVA, since the amplitude of an average is the same as the average of the amplitudes when there is an equal number of individual sweeps in the two conditions (and very nearly so for comparatively large numbers of sweeps). 

**We agree, but we take advantage of the format of this journal, which makes the previous versions available to the readers. They can see the results of both approaches.**

**Clerical:**

 In the abstract the word ‘inconsistent’ is split between two lines without hyphenation.  

**Corrected**

 Page 4, column 2, line 8: "if, and only if, that there is no way"  

**Corrected**

 In the Data Acquisition paragraph, only 26 of the 28 channels are listed, FP1 and FP2 present in the figures are missing here.  

**Corrected**

 Page 11, 2nd paragraph, “if” is missing in “Indeed, this were the case, one would have …”.  

**Corrected**

 Page 14, first paragraph, “of” is missing in “to verify the absence errors”.  

**Corrected**

 Many thanks.

# Peer review: Referee response for version 1

**DOI:** 10.5256/f1000research.6394.r7149

**Published:** 2015-01-07

**Authors:** André Achim

**Affiliations:** 1 Department of Psychology, Université du Québec à Montréal, Montréal, QC, Canada

The scientific study of consciousness is extremely difficult. Any serious attempt should therefore be regarded with a positively open mind. The intellectually challenging results of the present experiment, however, are unlikely to pertain to understanding the nature of qualia in the human brain. They rather seem to hint at a social cognition effect.

**Design**

The data consist in the average amplitude from 600 to 900 ms post stimulus onset of event related potentials (ERP) to complex stimuli selected as “most striking pictures” from the International Affective Picture System (IAPS). EEG recordings were obtained from dyads of socially related participants, simultaneously presented on separate halves of the same screen with a picture that could not be seen by the other participant, even though they were seated side by side and could see each other in the periphery of their visual field. Each participant had to observe the pictures in order to recognise them in a later memory test.

Four different ERP conditions are compared, each consisting of the average of 70 stimuli (minus occasional artefacts at some channels) obtained from the same block. Two of the blocks were introduced to the two participants to consist of identical stimuli simultaneously presented to each of them, and the remaining two blocks were announced to consist of different sets of pictures. In each pair of conditions, this initial announcement was faithful once and contrary to the actual situation the other time. This allowed analyzing the situation in a 2 x 2 design of Sameness (Identical versus different pairs actually presented) by Belief (believing that the partner was presented with same versus different stimuli).

The recordings, in each participant, were from 28 channels, referred to right ear lobe, not including any EOG dedicated channels. Each picture was presented for 1.0 s with a mean expected inter stimulus interval just below 1.15 s. Therefore, each of the four stimulus block lasted only about 2.5 minutes. The following simultaneous testing phase of the two participants required 28 minutes (280 targets + 280 new items each presented for 3.0 s).

Although the rationale for the study considers qualia and the possibility of common brain signatures, the averaging over different stimuli in the same condition restricts considerably the relevance of the data to the original question of the nature of the qualia and of their physical support in the brain. The authors actually need to extend the concept of qualia to include the knowledge of whether the other person in the dyad is supposed to receive identical or different stimuli, which is very unlikely the dominant subjective experience (qualia) produced by any stimulus. The data are therefore much more likely to pertain to social cognition than to the intended exploration of a physiological substrate for the qualia. Actually, for features of visual perception that do not play a role in the task (for instance by being rare versus frequent events or relevant versus irrelevant), the ERP differences are likely to be idiosyncratic, requiring special statistical technique to detect differences that take different forms in different brains (e.g. Buchsbaum and Fedio, 1969, 1970, for the ERP difference between geometrical patterns and three letter words made of the same number of dots).

ERP results.

Visual inspection of the averages shows that the ERP were negative, relative to the 200 ms baseline, for the full duration of the stimuli in all four conditions for all 12 channels anterior to Cz, and were entirely positive in 6 of the seven posterior channels, at the level of, or posterior to Pz, excluding Pz itself which is mostly negative up to about 400 ms and positive thereafter in all four conditions. Despite the large anterior-posterior inversion in overall ERP polarity, the amplitude differences between conditions remain constant in polarity across all channels.

The differences in condition are most marked between the two Believe Different conditions, with the Actually Same condition (SBd) being positive relative to the Actually Different condition (DBd). In the right hemisphere and posteriorly in the left hemisphere, the two Believe Same conditions (Actually Same and Actually Different, respectively SBs and DBs) were of intermediate amplitudes. In the frontal half of the left hemisphere, however, the ERP to the two Believe Same conditions overlap substantially with those of SBd, all three being less negative than DBd.

The lack of inversion of the condition differences suggests that the underlying dominant effect is unlikely a simple modulation of the dominant sources of these ERP. The left frontal distinction from the remaining ordering of conditions could indicate the presence of at least two sources modulated by the experimental conditions.

Although the data were analyzed with Belief and Sameness as factors, according to the experimental plan, it is relevant to decide whether the conditions SBs, SBd and DBs differ among themselves. Since the data are available, these could be tested. The following table gives the p value of all effects involving the three level Condition factor (DBd being excluded).

     effect                            Electrode group

                                 Sagittal       Para sagittal       Lateral

     Cond                      .774              .699                 .863

Cond x Hem                                     .353                 .228

Cond x Elect                .208              .128                 .489

Cond x Hem x Elect                          .066                 .131

Thus, even without correcting for these 10 tests involving Condition, there is no indication of any difference in this group of three conditions. Since there was no condition in which the participants were alone, it cannot be decided empirically whether the effect of watching stimuli in dyads affects essentially the DBd condition or the remaining three conditions.

The “operational hypothesis” that DBd would have a minimal impact on the ERPs implies that the topography in that condition is essentially that of the background activity. Since the background activity inverses polarity from front to back, this should also occur in the DBd condition. Since it does not, it becomes difficult to consider *de facto* DBd as the baseline no-effect condition. It could well be that DBd is the only condition that expresses the social cognition effect apparently present in the data.

Although *ad hoc*, the following explanation may be proposed for the present data in terms of social cognition. In the DBd condition, each partner might be interested in whether the other person is presented with a similar amount of emotion. This would also be so at the beginning of the SBd condition, but the impression would rapidly build that the stimuli, although believed different, would be matched for emotion and therefore this interest in the amount of emotion felt by the other person could fade. This would account for the SDd-DBd difference being the most reliably detected. For the two Believe Same conditions, each partner would not doubt that the other person receives the same amount of emotion, resulting in no difference between these conditions.

**Correlations**

The result section also includes correlations calculated for P600 amplitude differences between conditions. These are reported, in Table 4, only for “the conditions that were the most different from each other, namely, SBd-DBd and DBs-SBs”. This justification seems incorrect, given the above table showing no detected difference between SBd, DBs and SBs, and since additional tests of the latter pair does not show any difference at any channel (all p>=.073). Reproducing the scatter plots of Figure 7 confirms that only 14 intact dyads were actually retained (an odd numbered participant followed by the next even number in the data base provided; the rationale for rejecting some participants should have been expressed). The situation, however calls for using the intra class correlation coefficient (ICC) in which no distinction is made as to which member should be A and which should be B, and for which only the common sample mean and common sample variance are used, resulting in one extra degree of freedom for the test, since only one mean is fitted to the data. When ICC are calculated, the following can be obtained (the sign after the channel name duplicates that of the correlation).

ICC in **SBd**- **DBd**: 4 channels with p<.05

     9  r=-0.5674 t(13)=-2.4843 p=0.0274*  F8-

    17 r=-0.5438 t(13)=-2.3363 p=0.0361*  Fz-

    20 r=-0.6196 t(13)=-2.8462 p=0.0138*  P3-

    22 r=-0.6207 t(13)=-2.8545 p=0.0135*  Pz-

ICC in ICC in DBs- **DBd**: no channel with p<.05

ICC in ICC in *SBs*- **DBd**: no channel with p<.05

ICC in **SBd**- *SBs*:  1 channel with p<.05

    21 r= 0.6947 t(13)=3.4823 p=0.0040**  P4+

ICC in *SBs*- DBs: 3 channels with p<.05

    18 r= 0.5142 t(13)= 2.1617 p=0.0499*  O1+

    19 r= 0.5305 t(13)= 2.2562 p=0.0419*  O2+

    21 r= 0.6208 t(13)= 2.8551 p=0.0135*  P4+

The other condition differences were not tested. Note that 8/140 (perhaps not independent) correlations tested have p<.05, while 7/140 is expected for independent statistical tests when H0 is true. The critical value of p, with a Bonferroni correction (for 140 independent tests) would be .0018, not reached by any of the 140 tests.

Besides the possibility of there being no true correlation between the dyad members, the negative correlations between the dyad members, observed for the SBd-DBd difference, are counterintuitive. One could call upon disentanglement in which collapsing of the wave function for a particle causes the collapse of the complementary state, even at great distances. But here we have negative correlations on average amplitude differences. The quantum physics speculation not only would dispose of the phenomenon, assuming it is not a statistical accident, as being apparently explained, but this would require further *ad hoc *speculations to explain that the wave function would systematically collapse in the same way in the same person in the given test situation.

If there is a true phenomenon to understand, we should start by questioning whether the negative correlations are mostly associated with SBd or with DBd. Note that since the same variable is used for both members of the dyads, changing its sign would not alter the direction of the correlation. It is unlikely that DBd is the source of the negative correlations since no significant (p<.05) correlation is seen in any other difference involving DBd. But the source of the negative correlation is not likely to be SBd either since the other tested difference involving SBd does not replicate the negative correlations. While the negative correlations for SBd-DBd were significant (p<.02) at P3 and Pz, it is a positive correlation that is significant (p<.01) at P4 for SBd-SBs. Since P4 also gives a significant (p<.02) positive correlation for SBs-DBs, the positive correlation for SBd-SBs is more likely attributable to SBs than to SBd (which was involved in the negative correlation).

Setting aside the problem of ascribing the negative correlation to one of the involved experimental conditions, some important insight about social cognition could come from trying to identify the characteristics which defines which member of a dyad would produce a large or positive difference between conditions and which one would produce small or negative differences. Could this, for instance, characterize an implicit cognitive domination-submission attitude? But whether any of these correlations reflects a true correlation remains to be established first. The above discussion casts serious doubts on this.

**Technical details**

In reporting the behavioral data in Table 5, the misses do not need to be reported, as they should be 70 minus the number of hits (not exactly so here probably because of rounding errors), but the false alarm rate for the 280 new pictures should be added to allow estimating the amount of guessing.

In Figure 2, it is not clear to what the 20 cm distance applies. It would be relevant, however to know how far apart from each other were the participants and at what distance from the screen were their eyes, so we can appreciate how much they could see of each other.  Instead of the first and last half of participants, Figures 5 and 6 could provide the means form the participants on the left and those on the right (even though they are not the same in number), so that any tendency to gaze at the other person at some systematic time after stimulus delivery would be reflected by opposite shifts at F7 and F8. From the data available, there is no systematic group difference if F8-F7 in any or the four conditions, but that tells nothing of the 0-600 ms interval.

If systematic correlations existed between dyad members, the independence of participants in the ANOVA would not be achieved, so that the degrees of freedom would actually be inflated. This possible bias could be acknowledged, even though the presence of correlations is not very convincing.

On page 6: the statement “electrodes for which amplitude exceeded +/- 100 mV were discarded” would need clarification about the period of time in which this would be observed, since event exclusion was done channel by channel. Is that in the -200 to +1200 ms interval of an event?

In Figures 3, 5, and 6, the downward notch at the end of most tracings seems to be an artefact of filtering the average ERP. If these notches are artefacts, rather that brain responses, this should be explained.

The legend of Figure 4 gives the interval of interest as 600-1000 ms instead of 600-900 ms.

On page 11 in the Dataset box, remove “32”

I have read this submission. I believe that I have an appropriate level of expertise to confirm that it is of an acceptable scientific standard, however I have significant reservations, as outlined above.

# Response

**Published:** 2015-08-18

**Authors:** J. Bruno Debruille

**Affiliations:** McGill University, Canada

The scientific study of consciousness is extremely difficult. Any serious attempt should therefore be regarded with a positively open mind. The intellectually challenging results of the present experiment, however, are unlikely to pertain to understanding the nature of qualia in the human brain. They rather seem to hint at a social cognition effect.

**We acknowledge the possibility that the differences found might not pertain to qualia. We now make this clear at several places. For instance in** **the discussion, last two sentences of last paragraph of page 12 of the pdf, which reads:**

**“However, it is very important to point out that *nothing* in the present data can *directly* be related to qualia. The P600 effects of consistency only support spontaneous direct brain-to-brain influences that just *could* be related to consciousness.”**

**We thus, nevertheless, believe, particularly after our new statistical analyses revealing an effect of consistency at each of the three electrode subsets, that the results point to direct brain-to-brain communications, in addition to social cognition effects, which are now studied and analyzed to address this issue. They appear different from the ERP effects of consistency.**

**But, we do realize (and acknowledge in the paper) that the brain-to-brain communications might have nothing to do with consciousness. It has to be noted, however, that this possibility would then not be the most parsimonious, since it implies that qualia would have to be underlain by yet another phenomenon than the one responsible for the spontaneous brain-to-brain influences observed.**

**Design**

The data consist in the average amplitude from 600 to 900 ms post stimulus onset of event related potentials (ERP) to complex stimuli selected as “most striking pictures” from the International Affective Picture System (IAPS). EEG recordings were obtained from dyads of socially related participants, simultaneously presented on separate halves of the same screen with a picture that could not be seen by the other participant, even though they were seated side by side and could see each other in the periphery of their visual field. Each participant had to observe the pictures in order to recognize them in a later memory test.

Four different ERP conditions are compared, each consisting of the average of 70 stimuli (minus occasional artefacts at some channels) obtained from the same block. Two of the blocks were introduced to the two participants to consist of identical stimuli simultaneously presented to each of them, and the remaining two blocks were announced to consist of different sets of pictures. In each pair of conditions, this initial announcement was faithful once and contrary to the actual situation the other time. This allowed analyzing the situation in a 2 x 2 design of Sameness (Identical versus different pairs actually presented) by Belief (believing that the partner was presented with same versus different stimuli).

The recordings, in each participant, were from 28 channels, referred to right ear lobe, not including any EOG dedicated channels. Each picture was presented for 1.0 s with a mean expected inter stimulus interval just below 1.15 s. Therefore, each of the four stimulus block lasted only about 2.5 minutes. The following simultaneous testing phase of the two participants required 28 minutes (280 targets + 280 new items each presented for 3.0 s).

Although the rationale for the study considers qualia and the possibility of common brain signatures, the averaging over different stimuli in the same condition restricts considerably the relevance of the data to the original question of the nature of the qualia and of their physical support in the brain. The authors actually need to extend the concept of qualia to include the knowledge of whether the other person in the dyad is supposed to receive identical or different stimuli, which is very unlikely the dominant subjective experience (qualia) produced by any stimulus. The data are therefore much more likely to pertain to social cognition than to the intended exploration of a physiological substrate for the qualia. Actually, for features of visual perception that do not play a role in the task (for instance by being rare versus frequent events or relevant versus irrelevant), the ERP differences are likely to be idiosyncratic, requiring special statistical technique to detect differences that take different forms in different brains (e.g. Buchsbaum and Fedio, 1969, 1970, for the ERP difference between geometrical patterns and three letter words made of the same number of dots).

ERP results.

Visual inspection of the averages shows that the ERP were negative, relative to the 200 ms baseline, for the full duration of the stimuli in all four conditions for all 12 channels anterior to Cz, and were entirely positive in 6 of the seven posterior channels, at the level of, or posterior to Pz, excluding Pz itself which is mostly negative up to about 400 ms and positive thereafter in all four conditions. Despite the large anterior-posterior inversion in overall ERP polarity, the amplitude differences between conditions remain constant in polarity across all channels.

The differences in condition are most marked between the two Believe Different conditions, with the Actually Same condition (SBd) being positive relative to the Actually Different condition (DBd). In the right hemisphere and posteriorly in the left hemisphere, the two Believe Same conditions (Actually Same and Actually Different, respectively SBs and DBs) were of intermediate amplitudes. In the frontal half of the left hemisphere, however, the ERP to the two Believe Same conditions overlap substantially with those of SBd, all three being less negative than DBd.

The lack of inversion of the condition differences suggests that the underlying dominant effect is unlikely a simple modulation of the dominant sources of these ERP. The left frontal distinction from the remaining ordering of conditions could indicate the presence of at least two sources modulated by the experimental conditions.

Although the data were analyzed with Belief and Sameness as factors, according to the experimental plan, it is relevant to decide whether the conditions SBs, SBd and DBs differ among themselves. Since the data are available, these could be tested. The following table gives the p value of all effects involving the three level Condition factor (DBd being excluded).

     effect                            Electrode group

                                 Sagittal       Para sagittal       Lateral

     Cond                      .774              .699                 .863

Cond x Hem                                     .353                 .228

Cond x Elect                .208              .128                 .489

Cond x Hem x Elect                          .066                 .131

Thus, even without correcting for these 10 tests involving Condition, there is no indication of any difference in this group of three conditions. Since there was no condition in which the participants were alone, it cannot be decided empirically whether the effect of watching stimuli in dyads affects essentially the DBd condition or the remaining three conditions.

The “operational hypothesis” that DBd would have a minimal impact on the ERPs implies that the topography in that condition is essentially that of the background activity. Since the background activity inverses polarity from front to back, this should also occur in the DBd condition. Since it does not, it becomes difficult to consider *de facto* DBd as the baseline no-effect condition. It could well be that DBd is the only condition that expresses the social cognition effect apparently present in the data.

Although *ad hoc*, the following explanation may be proposed for the present data in terms of social cognition. In the DBd condition, each partner might be interested in whether the other person is presented with a similar amount of emotion. This would also be so at the beginning of the SBd condition, but the impression would rapidly build that the stimuli, although believed different, would be matched for emotion and therefore this interest in the amount of emotion felt by the other person could fade. This would account for the SDd-DBd difference being the most reliably detected. For the two Believe Same conditions, each partner would not doubt that the other person receives the same amount of emotion, resulting in no difference between these conditions.

**Many of the above comments end up casting doubts on the choice of the Different Believed-different (DBd) condition as a baseline condition. We acknowledge that this choice is in itself debatable. On the other hand, the differences obtained between this condition and the others were small (in the order of a microvolt) whereas standard deviations were in the order of 3.5 microvolts. Also, like the other conditions, DBd, was itself a bit noisy. Thus, it could have differed from the other conditions by chance. This could have happened because of the diversity of IAPS stimuli and because there was no speeded decision to make at each trial, which is known to produce sub-optimal ERPs.**

**One way to deal with these problems was to further improve the signal to noise ratio by doubling the number of trials per condition and thus, by grouping conditions.**

**This was doable, since after all, to test the fundamental hypothesis that there is some direct brain-to-brain communication that might have an effect on P600s, all that was needed was to see whether conditions that are actually consistent with the belief differed from the conditions where the stimulus displayed to the other participant was not consistent with belief. This is what we decided to do. Therefore, in each participant, we averaged together SBs with DBd (=conditions consistent with the belief) and DBs with SBd (=belief inconsistent conditions).**

**The new grand averages are shown in the new Fig, 2. The ERPs look less noisy (still without any smoothing) and visual inspection suggests differences between consistency and inconsistency.**

**Statistical analyses confirmed the significance of these ERP differences (see new results section). Because it was impossible for each participant to see and check whether the stimulus presented to his/her partner was consistent with the belief, which would have been the only classical means by which those differences could have been observed, then, we can say that these results support the existence of direct brain-to-brain communications.**

**The possibility that the emotional reaction of the other participant to the stimulus could develop, be detected/processed by the subject and impact P600s was extremely unlikely. The time would be too short for the brain of the 1 ^st^ participant to analyze the stimulus, to organize an appropriate emotional output to the muscles, then, for the muscles to actually move, for the other participant to detect these moves in his/her very peripheral visual-field, to process them and to detect their inconsistency with his own emotion in 600 ms.**

****

**To further strengthen this timing argument, we used the small ERP differences that can be detected by the visual inspection of Fig, 2 in the N450 time window, especially at frontal electrode sites (e.g., Fz). We analyzed them. They were significant (now in the new results section), suggesting that the timing was even shorter (about 400 ms), further discarding the emotional account.**

**These new results are now used in the discussion to support the existence of direct brain-to-brain communications.**

**The part of the reviewer’s comment that we underlined is now addressed by computing the ERPs according to “ whether the other person in the dyad is supposed to receive identical or different stimuli”**

**New statistical analyses were run to test the significance of the small ERP differences found at left anterior electrode scalp site. They were significant, but the scalp distribution of these social cognition effects was quite different from that of the consistency effect (see below).**

**Correlations**

The result section also includes correlations calculated for P600 amplitude differences between conditions. These are reported, in Table 4, only for “the conditions that were the most different from each other, namely, SBd-DBd and DBs-SBs”. This justification seems incorrect, given the above table showing no detected difference between SBd, DBs and SBs, and since additional tests of the latter pair does not show any difference at any channel (all p>=.073). Reproducing the scatter plots of Figure 7 confirms that only 14 intact dyads were actually retained (an odd numbered participant followed by the next even number in the data base provided; the rationale for rejecting some participants should have been expressed). The situation, however calls for using the intra class correlation coefficient (ICC) in which no distinction is made as to which member should be A and which should be B, and for which only the common sample mean and common sample variance are used, resulting in one extra degree of freedom for the test, since only one mean is fitted to the data. When ICC are calculated, the following can be obtained (the sign after the channel name duplicates that of the correlation).

ICC in **SBd**- **DBd**: 4 channels with p<.05

     9  r=-0.5674 t(13)=-2.4843 p=0.0274*  F8-

    17 r=-0.5438 t(13)=-2.3363 p=0.0361*  Fz-

    20 r=-0.6196 t(13)=-2.8462 p=0.0138*  P3-

    22 r=-0.6207 t(13)=-2.8545 p=0.0135*  Pz-

ICC in ICC in DBs- **DBd**: no channel with p<.05

ICC in ICC in *SBs*- **DBd**: no channel with p<.05

ICC in **SBd**- *SBs*:  1 channel with p<.05

    21 r= 0.6947 t(13)=3.4823 p=0.0040**  P4+

ICC in *SBs*- DBs: 3 channels with p<.05

    18 r= 0.5142 t(13)= 2.1617 p=0.0499*  O1+

    19 r= 0.5305 t(13)= 2.2562 p=0.0419*  O2+

    21 r= 0.6208 t(13)= 2.8551 p=0.0135*  P4+

The other condition differences were not tested. Note that 8/140 (perhaps not independent) correlations tested have p<.05, while 7/140 is expected for independent statistical tests when H0 is true. The critical value of p, with a Bonferroni correction (for 140 independent tests) would be .0018, not reached by any of the 140 tests.

Besides the possibility of there being no true correlation between the dyad members, the negative correlations between the dyad members, observed for the SBd-DBd difference, are counterintuitive. One could call upon disentanglement in which collapsing of the wave function for a particle causes the collapse of the complementary state, even at great distances. But here we have negative correlations on average amplitude differences. The quantum physics speculation not only would dispose of the phenomenon, assuming it is not a statistical accident, as being apparently explained, but this would require further *ad hoc *speculations to explain that the wave function would systematically collapse in the same way in the same person in the given test situation.

If there is a true phenomenon to understand, we should start by questioning whether the negative correlations are mostly associated with SBd or with DBd. Note that since the same variable is used for both members of the dyads, changing its sign would not alter the direction of the correlation. It is unlikely that DBd is the source of the negative correlations since no significant (p<.05) correlation is seen in any other difference involving DBd. But the source of the negative correlation is not likely to be SBd either since the other tested difference involving SBd does not replicate the negative correlations. While the negative correlations for SBd-DBd were significant (p<.02) at P3 and Pz, it is a positive correlation that is significant (p<.01) at P4 for SBd-SBs. Since P4 also gives a significant (p<.02) positive correlation for SBs-DBs, the positive correlation for SBd-SBs is more likely attributable to SBs than to SBd (which was involved in the negative correlation).

Setting aside the problem of ascribing the negative correlation to one of the involved experimental conditions, some important insight about social cognition could come from trying to identify the characteristics which defines which member of a dyad would produce a large or positive difference between conditions and which one would produce small or negative differences. Could this, for instance, characterize an implicit cognitive domination-submission attitude? But whether any of these correlations reflects a true correlation remains to be established first. The above discussion casts serious doubts on this.

**Response: We agree, and given, that these correlations**

1. **were not based on *a priori* hypothesis,**
2. **rested on single rather than on the new grouped conditions and **
3. **were not critical to discuss the possibility of a direct brain-to-brain communications, **

**we decided to remove them.** **Thus, they are not in the new version of the paper.**

**As to the comment:**

“The quantum physics speculation not only would dispose of the phenomenon, assuming it is not a statistical accident, as being apparently explained, but this would require further *ad hoc *speculations to explain that the wave function would systematically collapse in the same way in the same person in the given test situation”,

**We do not see why this systematicity would be mandatory for the system to detect inconsistency. It seems to us that this could happen in a particular way at each trial. Nevertheless, a real answer to this comment would involve physics far beyond our competences. For instance, what if being closely related to someone depends on some quantum entanglement, as audaciously proposed by some authors? We contacted Basil Hiley. He globally supported the paper but did not comment these issues. We tentatively imagine that the two entangled partners could act on quantum field energy in a similar way when seeing the same stimuli and knowing it and act on that field in different ways when seeing different stimuli while being told they are looking at the same.**

**We know that anything that is produced by a biological organism tend to be feedback regulated. Accordingly, the brain could be sensitive to some changes in the quantum field energy. It might then also capture something of the effect of the entangled partner on that field. This could lead to inconsistency detection whatever the pattern of the change, provided that this pattern differs from the one expected.**

**Could that be plausible? We would like to have expert opinions. We hope the paper will be of interests to physicists. However, many of them seem to adhere at Tegmar** **k’** **s rebuttal of the link between consciousness and quantum field energy (as the one made by Penrose). They no longer discuss such link.**

**Technical details**

In reporting the behavioral data in Table 5 (now Table 3), the misses do not need to be reported, as they should be 70 minus the number of hits (not exactly so here probably because of rounding errors), but the false alarm rate for the 280 new pictures should be added to allow estimating the amount of guessing.

**Response: We believe we have to disagree. The amount of guessing for new stimuli is irrelevant since it cannot be used to differentiate the two belief conditions of the study phase. The aim of this memory test was not classical. It was to test the hypothesis of a different encoding in memory according to the conditions of the study phase.**

In Figure 2, it is not clear to what the 20 cm distance applies. It would be relevant, however to know how far apart from each other were the participants and at what distance from the screen were their eyes, so we can appreciate how much they could see of each other. 

**Response: Thanks a lot for pointing to that figure. There were errors.**

**The distance from the eyes to the screen was about 60 cm. The piece of cardboard separating the two halves of the screen was 41cm long. The distance between the heads of the 2 participants of a pair (i.e., from the right ear of the participant on the left to the left ear of the participant on the right) was at about 40 cm. We replaced Figure 1 by a photo on which these distances were added, as well as the visual angle corresponding to the width of the stimuli used.**

Instead of the first and last half of participants, Figures 5 et 6 could provide the means form the participants on the left and those on the right (even though they are not the same in number), so that any tendency to gaze at the other person at some systematic time after stimulus delivery would be reflected by opposite shifts at F7 and F8. From the data available, there is no systematic group difference if F8-F7 in any or the four conditions, but that tells nothing of the 0-600 ms interval.

**Great idea. We did it. Now Fig 5 A & B. And, I did not support the idea of opposed eye movement (included in the results section).**

If systematic correlations existed between dyad members, the independence of participants in the ANOVA would not be achieved, so that the degrees of freedom would actually be inflated. This possible bias could be acknowledged, even though the presence of correlations is not very convincing.

**We agree. We now mention that brain–to-brain communications jeopardize the independence of participants and that the degrees of freedom could actually be inflated. This is now in the discussion, p 12, 2 ^nd^ paragraph, starting line 7.**

On page 6: the statement “electrodes for which amplitude exceeded +/- 100 mV were discarded” would need clarification about the period of time in which this would be observed, since event exclusion was done channel by channel. Is that in the -200 to +1200 ms interval of an event?

**Thanks for that too. There was a 1000-1200 ms non-reject interval. So trials were amplitudes outside of the+/-100 microvolts were within this late time window were kept. This is now mentioned (p6, last paragraph of 1st column, line 6).**

In Figures 3, 5, and 6, the downward notch at the end of most tracings seems to be an artefact of filtering the average ERP. If these notches are artefacts, rather that brain responses, this should be explained.

The legend of Figure 4 gives the interval of interest as 600-1000 ms instead of 600-900 ms.

**This downward notch at the end of most tracings correspond to the off-effect (i.e., the disappearance of the IAPS picture used as a stimulus), which might also be associated to an eye movement. This is now mentioned in the legend of Fig 2 and 3. We do not think it could be an artifact of filtering. It was probably due to the 1000-1200 ms non-rejection time window.**

On page 11 in the Dataset box, remove “32”

**Done. Note that this data set now includes the tables for the social-cognition (belief) effect together with that for the consistency effect within the P600 time-window and that for this effect in the N400 time window.**
